# Systemic risk mitigation in supply chains through network rewiring

**DOI:** 10.1038/s41598-026-42549-1

**Published:** 2026-03-05

**Authors:** Giacomo Zelbi, Leonardo Niccolò Ialongo, Stefan Thurner

**Affiliations:** 1 Complexity Science Hub Vienna, 1030 Vienna, Austria; 2 Supply Chain Intelligence Institute Austria, 1030 Vienna, Austria; 3 Medical University of Vienna, 1090 Vienna, Austria; 4 Santa Fe Institute, Santa Fe, NM 85701 USA

**Keywords:** Production networks, Systemic risk, Supply chain rewiring, Resilience, Monte Carlo simulation, Mathematics and computing, Physics

## Abstract

The networked nature of supply chains makes them susceptible to systemic risk, where local firm failures can propagate through interdependencies and lead to cascading supply chain disruptions. The systemic risk of supply chains can be quantified and is closely related to the topology and dynamics of supply chain networks (SCN). However, how different network topologies contribute to this risk remains unclear. Here, we ask whether systemic risk can be significantly reduced by rewiring supplier-customer pairs. In doing so, we quantify the extent to which the observed systemic risk is a result of fundamental properties of the dynamical system. We minimize systemic risk by employing a method from statistical physics that respects firm-level constraints to production. Analyzing six specific subnetworks of the national SCNs of Ecuador and Hungary, we demonstrate that systemic risk can be considerably mitigated by 16-50% without reducing the production output of firms. A comparison of network properties before and after rewiring reveals that this risk reduction is achieved by changing the connectivity in non-trivial ways. These results suggest that actual SCN topologies carry unnecessarily high levels of systemic risk and that resilience can be substantially enhanced by targeted supplier changes comparable in scale to one year of natural network evolution.

## Introduction

Supply chain networks (SCN) are dynamical socio-economic systems that arise from the spontaneous self-organization of companies transforming sets of inputs into goods and services. In an SCN, nodes represent companies that act as central decision units, determining what to produce, how to produce it, from whom to source inputs, and to whom and at what price to sell outputs. The outcomes of these decisions are temporary supply links that ensure firms have access to the necessary inputs and consumers for their products. A weighted directed link in the SCN, $W_{ij}$, indicates the quantity of a product supplied by firm *i* to firm *j*. While SCNs are to some extent formed by local optimizing behavior of firms and can be assumed to form locally robust structures with respect to small shocks, they remain vulnerable to larger and systemic supply disturbances. A temporary failure of a company, for example, can trigger disruptions that propagate through the network, amplifying the initial shock to systemically relevant levels and leading to considerable production and delivery disruptions^1,2^. Recent and historical events have exposed these fragilities, shaking the assumptions about the stability and reliability of SCNs. SCN disruptions have been studied in the context of natural disasters, such as the 2011 Thai floods^3^ or the earthquakes in Japan^4,5^. More recently, lockdowns and other measures during the COVID-19 pandemic have caused unprecedented SCN disruptions worldwide^6–8^ with long-lasting effects on prices and inflation^9^. The pandemic caused strong pressures on global supply chains, resulting in, for example, congestion at U.S. West Coast shipping terminals^10^ and shortages of semiconductor chips for the automotive industry^11^. Since 2022, geopolitical tensions have led to severe shortages in gas and grain supplies from Russia and Ukraine^12,13^. In times of changing geopolitical equilibria, the supply of raw materials and technologies across borders is becoming a source of major concern^14–16^.

Traditionally, the study of risks related to supply chain dependencies has been constrained by data limitations. Its focus has been on the aggregate sector-level dynamics (input-output analysis) and on focal firms and their direct suppliers and customers, only sometimes including information on multiple tiers. Recent advances in data availability of firm-level supplier-buyer relations offer unprecedented insights into supply chain dynamics^17,18^. While traditional input-output analysis, which aggregates firms into industrial sectors, fails to capture the heterogeneous nature of company-specific input and output patterns and thus neglects network structures, firm-level data reveal the underlying networks at the scale at which actual business decisions are made^19^. This new generation of data demonstrated the importance of detailed network effects in the propagation of production losses by comparing the results of a traditional sector-level scenario with the situation in a real SCN at the firm level^20^.

A key novelty of firm-level data is that they allow the estimation of the consequences of single-firm failures and assess their potential to trigger cascading disruptions^21,22^. In other words, it enables the quantification of the systemic risk contribution of individual firms within an economy. Systemic risk emerges from an interplay of the topology of interactions between economic actors, balance sheets, inventories, and the firms’ ability to replace their suppliers. Network-based systemic risk has been a subject of academic study in the context of financial markets, in particular, interbank lending markets^23–26^. Recently, this approach has been adapted and generalized for supply chains^21^. There, a firm’s systemic importance is quantified by the Economic Systemic Risk Index (ESRI), defined as the fraction of the total production of the economy affected by the failure of that firm. This quantification allows firms to be ranked by their systemic risk contribution (ESRI value), creating “systemic risk profiles” of economies. The ESRI profile computed for all Hungarian firms in the national SCN reveals how systemic risk is distributed across the economy and where it is concentrated. Notably, firms with large systemic risk contributions are limited to a tiny subset of businesses and are not necessarily the largest in size or revenue. In contrast to the static view assumed by most models, the SCN is continuously evolving, with companies rewiring their supply connections and firms entering and exiting the network. Evidence shows that approximately 55% of all supply relationships present at any given time will disappear within a year^27^. SCNs reshape their structure in response to shifting economic conditions such as innovations, price changes, and the sudden unavailability of suppliers exiting the market. When a supplier becomes unavailable, a company may respond in many ways, depending on its production function: it can relink to a new supplier, reduce its output, or rely on existing stock inventories. This raises a fundamental question: how does systemic risk vary with the changing network structure? Is it possible to find solutions within the space of network configurations that are associated with lower systemic risk? And to what extent can systemic risk be mitigated by strategically rewiring supplier-customer links? The substantial level of temporal rewiring of SCNs suggests that changes to the network structure are feasible. If the correct incentives were put in place, the system could indeed evolve towards lower systemic risk values with minimal intervention.

Here, we explore the potential for systemic risk mitigation in SCN topologies by modifying the network structure of interactions between firms, while maintaining production levels and functions. To this end, we propose a simple link-rewiring algorithm that preserves the production constraints of individual firms, and we apply it to real nationwide firm-level supply chain data. In particular, we investigate the extent to which systemic risk can be reduced in six subSCNs, extracted from the countrywide SCNs of Ecuador and Hungary. In this work, we abstract from the microeconomic mechanisms underlying supply-chain network formation and from firms’ supplier–customer selection decisions. Instead, we focus on identifying alternative network configurations that are compatible with the empirically observed production constraints. This approach allows us to assess the potential for systemic risk mitigation implied by these constraints, without addressing the costs or incentives associated with network reorganization. Our approach is based on the Metropolis-Hastings algorithm, which uses importance sampling in Monte Carlo simulations to choose link modifications that lower systemic risk while avoiding getting stuck in local minima of the systemic risk “landscape”; see Materials and Methods. A comparison between the systemic risk profiles of the optimal network configurations and those from the original network then allows us to quantify the potential for reducing systemic risk and identify which companies are responsible for this reduction. We then test whether the systemic risk reduction potential depends on specific properties of the network and firm.

**Fig. 1:**
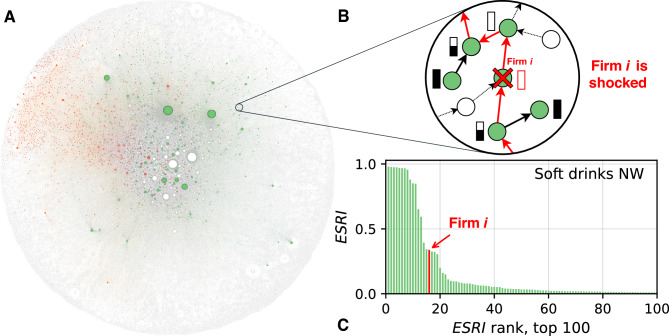
(**A**) Ecuadorian network comprised of 65,614 firms and 650,931 supply links. The crustaceans and soft drinks subnetworks are highlighted by red and green nodes. (**B**) Schematic view of a shock propagation event along the SCN after the default of a particular firm *i*. Suppliers and customers of firm *i* have to adjust and reduce their production (full bars indicate 100% production level). Note that the shock propagates up- and downstream the SCN, affecting the firms’ production levels represented by the bars. (**C**) Systemic risk profile. The panel shows the rank distribution of the systemic risk contributions of all firms (ESRI value) in the soft drinks SCN.

The idea of finding networks with minimal systemic risk levels has been explored in the context of financial networks. Previous studies have examined the effect of taxes on interbank links that create systemic risk, demonstrating a significant mitigation potential of up to 50%^28,29^. In a different approach, interbank lending networks were optimized by employing a Mixed-Integer Linear Programming algorithm with the constraint that banks’ total assets and liabilities remained untouched. Again, the rewired interbank networks showed a significant reduction of systemic risk of about 70%^30^. Another work focused on portfolio optimization to reduce systemic risk arising from overlapping bond exposures in European banking stress-test data^31^. However, these approaches cannot be directly applied to supply chain data, as supply interactions between firms differ fundamentally from financial exposure. Unlike financial networks, SCNs must adhere to production constraints, geographical limitations, and technological compatibility requirements, introducing additional layers of dynamics.

To evaluate the systemic risk content of a given SCN, we use the Economic Systemic Risk Index (ESRI) as proposed in^21^. The ESRI of firm *i* quantifies the proportion of the total production lost due to demand and supply disruptions propagating up and down through the SCN following the failure of firm *i*. An example of such a cascade is shown in Fig. 1B, where we zoom in on the neighborhood of firm *i*, in the Ecuadorian national SCN depicted in Fig. 1A. The disruption propagates upstream and downstream, via the directed links connected to *i*. The firms (represented as circles) that depend on *i* for inputs experience shortages and scale down their production, while suppliers to *i* reduce their production in response to the loss of a customer. The cascade propagates and affects second-tier firms and so on. ESRI$_i$ is the fraction of total production remaining after the shock has spread compared to the total production before the default of *i*. Note that ESRI takes into account the heterogeneous nature of production functions (firms can have essential or non-essential inputs), as well as the replaceability of suppliers. Computing ESRI for all firms in the system yields the risk profile of the SCN, as shown in Fig. 1C. To quantify the overall systemic risk of the SCN, we use the average ESRI across all firms in the SCN, $\langle \text {ESRI}\rangle$. For further details on ESRI, see Materials and Methods, SI section S2 and^21^.

### Constraints for the rewiring algorithm

To estimate the potential for systemic risk mitigation through rewiring, it is necessary to determine which network configurations are allowed. Not all possible connections between firms are meaningful or feasible – most are not. The objective of our algorithm is to maintain the system in its current state, except for a number of firms changing suppliers. We therefore do not change the production technology of each firm, nor its capacity. This means preserving the empirical technical coefficient given by the ratio of intermediate input per unit of output. We do so by making sure that, for each firm, the total output and input per product remain close to their empirical values. In practice, we allow for a maximum change in total production output of 20%, while keeping the input quantities per product unchanged. In addition, since connections are costly to establish, and given the relation between a firm’s connectivity and its systemic importance (see SI section S7), we do not want to significantly change the number of suppliers the firm has for each of its intermediate inputs. To guarantee this, we employ an algorithm based on link swapping that ensures that a supplier is replaced by another firm providing the same product or service. For further details on the rewiring steps, see SI section S3.

**Fig. 2:**
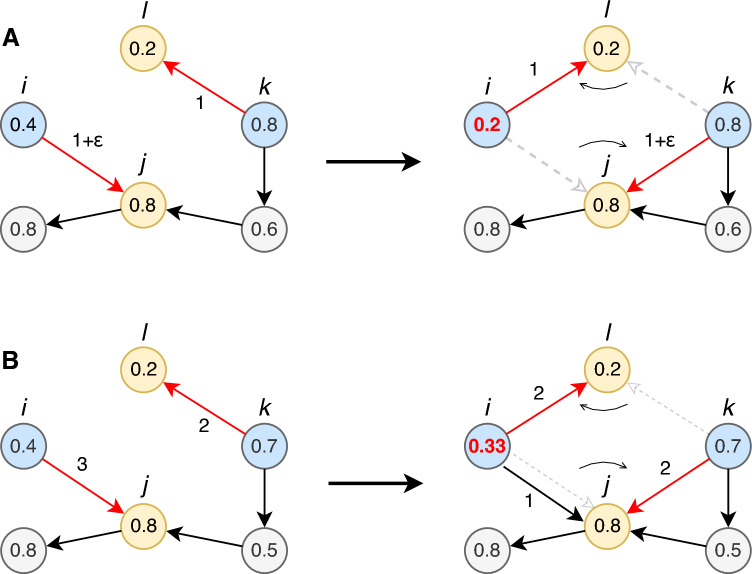
Schematic example of a constrained network rewiring step. Node color represents the economic activity (a proxy for the goods manufactured or services provided) and the numbers inside the nodes indicate their contribution to systemic risk, ESRI. At every rewiring iteration, two directed supply links are randomly selected, ensuring they share the same ordered combination of economic activities at their source and target nodes. In this example (left side of panels), the selected links (red arrows) are $i\rightarrow j$ and $k\rightarrow l$, both connecting blue nodes to yellow nodes. (**A**) If the two links have similar weights within a set tolerance threshold, the rewiring step (fat black arrow) swaps the suppliers of the target nodes *j* and *l*. As a result, *i* now sells to *l*, and *k* sells to *j* (right side). A constraint prevents small weight differences from accumulating over multiple swaps. (**B**) If the difference in the link weights exceeds the tolerance threshold, the link with the larger weight is split, and only the portion matching the smaller weight is swapped. After rewiring, the topology of the production network changes, and so does the failure cascade following a local shock, with ESRI values changing accordingly for some nodes. Here node *i* now has decreased its ESRI; the difference is only due to the change in topology.

To explore the space of supply chains that satisfy these constraints, we propose a Monte Carlo link-swapping algorithm. A schematic representation is shown in Fig. 2. At every time step, we randomly select two supply links (red arrows, $i \rightarrow j$ and $k \rightarrow l$) that connect companies with the same pair of economic activities, indicated by colors in the figure (e.g., blue sells to yellow, with matching colors at the source and target). If the two links have the same weight (as in panel A), we swap them entirely. This swap (fat black arrow) can be interpreted as exchanging the suppliers of the target nodes or the customers of the source nodes. However, since supply link weights represent product volumes, it is unlikely that two links will have exactly the same weight. To address this problem, we introduce a small tolerance by allowing full swaps when weights differ by a value of $\epsilon$ or less. To prevent unintended accumulation of changes, we impose a constraint that ensures that a firm’s total out-strength does not deviate by more than 20% from its original value. Our results are robust with respect to increasing or decreasing this threshold as we show in SI section S3. Note that this swap does not change the overall degree distributions in the SCN. If instead the difference in link weights is too large for a full swap, the link with the larger weight is split, and only a portion equal to the smaller weight is exchanged (as shown in panel B). This introduces a new link but preserves the technical coefficients of all firms involved. If no weight information is available, all links can be considered of the same weight, and the algorithm operates as in case A. All swaps are reversible, ensuring free exploration of configuration space.

Even under these strict constraints, for realistic SCN sizes, the number of possible network configurations is immense, and computing the average systemic risk of every configuration is not feasible. Therefore, we sample them using a Metropolis-Hastings algorithm with an acceptance criterion that biases the link-rewiring moves toward lower-risk configurations. After every swap iteration, we compute the average ESRI for the new trial SCN. If the $\langle \text {ESRI}\rangle$ in the trial configuration is lower, the swap is accepted; otherwise, if the trial configuration leads to a higher $\langle \text {ESRI}\rangle$, the swap is accepted only with a probability $p = \exp (-\beta (\langle \text {ESRI}\rangle _{after}-\langle \text {ESRI}\rangle _{before}))$. If the swap is rejected, we continue with the next swap iteration. The parameter $\beta > 0$ is sometimes called an “inverse temperature”, and it controls the likelihood of accepting moves that increase $\langle \text {ESRI}\rangle$, helping the system to escape local minima. By gradually increasing $\beta$ over swap iterations, the system converges toward an optimal configuration (simulated annealing). To calibrate these increasing $\beta$ curves, we first run simulations at fixed $\beta$. See Materials and Methods for more details.

## Results

We apply the rewiring algorithm to six empirical subnetworks to assess its impact on network resilience, across different production networks. From Ecuador’s 2015 SCN, we analyze two food industry subnetworks: the processing of fish, crustaceans and molluscs network and the manufacturing of soft drinks network. From Hungary’s 2017 SCN, we extract the unweighted networks for food production and the automotive industry. Each subnetwork comprises approximately 1,000 nodes and fewer than 10,000 supply links, making $\langle \text {ESRI} \rangle$ computation feasible at every swap iteration. For details on subnetwork extraction, see SI section S1. In the VAT datasets there is no information on the products being exchanged. Therefore, we choose the NACE 3-digit classification^32^ of the supplying firms as a proxy for the product classification of the link used in the computation of ESRI. Note that the swapping algorithm not only guarantees that the amount of inputs by product used by each firm does not change, but also guarantees that the output by receiving industry is not modified. This further guarantees that the input-output structure of the network is not altered.

**Fig. 3:**
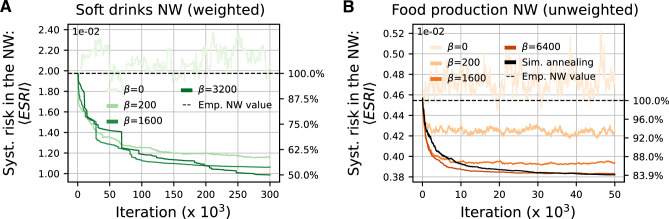
Decrease of systemic risk, $\langle \text {ESRI}\rangle$, as a function of rewiring (Monte Carlo) steps for (**A**) the soft drinks subnetwork of Ecuador (2015) and (**B**) the food production subnetwork of Hungary (2017), the latter representing an unweighted SCN. Results are shown for different values of the “inverse temperature”, $\beta$. For $\beta =0$, the “configuration model”, every rewiring step is accepted and, interestingly, the systemic risk level remains close to the empirical value. For larger $\beta$, the system quickly converges to plateaus of lower-risk network configurations, with the final risk level depending on $\beta$. While a higher $\beta$ generally leads to lower and greedier minima convergence, it also increases the likelihood of getting trapped in local ones. This is particularly evident for $\beta =3200$ in A, where several times plateaus form, although occasionally a lucky swap explores a new local minimum. In the unweighted case B this effect is less pronounced. To ameliorate this problem, we employ the simulated annealing method (black lines). The empirical systemic risk values (initial level) are shown as the dashed line. The simulations are stopped after 300,000 and 50,000 iterations for the soft drinks and food production networks, respectively.

We iteratively rewire each subnetwork, starting from the true empirical configuration. Figure 3 shows the average $\langle \text {ESRI}\rangle$ as a function of rewiring steps, for (A) the soft drinks network and (B) the unweighted food production network. Colored curves represent different fixed-$\beta$ rewiring simulations, and the black lines correspond to simulated annealing ($\beta$-increasing). The dashed line indicates the systemic risk level of the empirical subnetworks. For $\beta =0$ (accepting every swap regardless of risk impact), the systemic risk remains around the empirical level, indicating that without intervention, SCNs tend to settle in suboptimal configurations far from minimal systemic risk. The $\beta =0$ case is sometimes referred to as “configuration model”.

For higher $\beta$ values, the simulations converge to different plateaus of low-risk network configurations after several tens of thousands of rewiring steps. Notably, 10,000 steps roughly correspond to one update for each supply link, equivalent to less than two years of real-world rewiring rates observed in the Hungarian SCN^27^. The choice of (fixed) $\beta$ seems to select the systemic risk level at convergence, with higher values generally leading to lower risk. However, excessively high $\beta$ increases the chance of getting trapped in local minima. In some cases, the algorithm still finds a move that allows the system to escape, triggering a sharp drop in systemic risk, as seen for $\beta =3200$ in Fig. 3A. To find a balance between finding lower plateaus and the risk of getting stuck, simulated annealing varies $\beta$ throughout the simulation (see Materials and Methods), typically yielding the best results. While these results are likely not global minima, they provide a reasonable estimate of the achievable level of risk reduction. For simulations with different $\beta$ values and other networks, see SI section S4. In summary, the systemic risk reduction varies across the networks, ranging from 16% to 50%, as summarized in Table 1 for the different production networks.

**Fig. 4:**
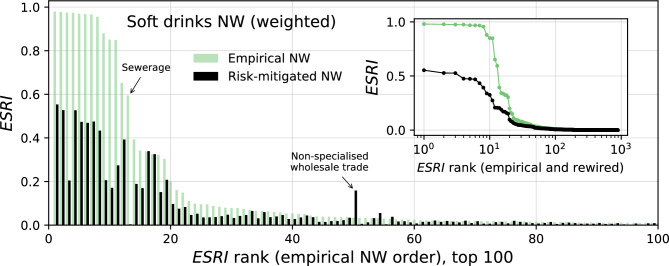
Systemic risk profile of the empirical (light green bars) and risk-mitigated (black bars) soft drinks network. Firms are ranked by their ESRI values in the empirical network along the *x*-axis, displaying only the top 100 riskiest firms. Rewiring reduces the systemic risk contribution of most firms, with a few exceptions where risk increases (as for a firm in *Non-specialised wholesale trade*). The largest reductions occur among the highest-risk firms, while the systemic risk contribution of the distribution’s tail does not change significantly after rewiring. The inset presents the same profiles, with the rewired profile now rank-ordered for direct comparison.

We find that risk mitigation through relinking is nearly as effective for unweighted networks (Fig. 3B), suggesting that network topology is at the heart of systemic risk, while link weights play only a secondary, though important, role. We also note that convergence appears to be substantially faster for unweighted networks, likely because random swaps frequently target links of negligible importance, of which there are many due to the fat-tailed distribution of link weights. To further examine the role of weights, we constructed unweighted versions of the Ecuadorian soft drinks and crustaceans networks and compared the results of the rewiring algorithm (Table 1). The initial average ESRI values for the unweighted case closely match those of the crustaceans network but differ significantly for the soft drinks network, where the empirical ESRI values are nearly twice as high in the weighted version. This could be due to several factors, one hypothesis being the substitutability of firms that depends on the market share of the firm in a given product. The weighted information significantly alters this market share and can therefore lead to an increase in systemic relevance of firms. In terms of risk mitigation potential, the crustaceans network again shows similar results between weighted and unweighted cases, whereas in the soft drinks network, the weighted version achieves a higher risk reduction. A comparison of the risk profiles of the weighted and unweighted crustaceans subnetworks (see SI section S5) yields that, in the unweighted case, the risk is concentrated within a very small set of firms, just 5 compared to the 11 in the weighted case. It seems that when the risk is so concentrated in a few firms, the algorithm cannot find possible moves for significant risk reduction. Note that also in the other case of a smaller reduction, the Hungarian food subnetwork, the risk is mainly due to two firms, which suggests that in these cases the production constraints do not allow further mitigation.

Rewiring severely affects the systemic risk profile, i.e., the rank-ordered distribution of firms, as shown in Fig. 4. The top 100 riskiest firms in the Ecuadorian soft drinks production network are compared before (light green bars) and after applying the rewiring algorithm with simulated annealing (black bars). The ESRI values of the riskiest firms shrink considerably, often by 50% or more. For one firm (in NACE 37.0 - sewerage), it drops to almost zero. Only a few firms increase their ESRI after rewiring, as the one indicated by the arrow (NACE 46.9 - Non-specialized wholesale trade). The inset shows the same systemic risk profiles, now with the rewired profile also rank-ordered. Similar results for other networks are shown in SI section S5.

To find out how rewiring alters the structural properties of SCNs beyond ESRI values, we compare several network metrics before and after rewiring in Table 1. There are only a few notable changes to these measures. As expected, the weighted rewiring tends to introduce new links rather than aggregate them. Interestingly, the increase in the number of links, *L*, does not correlate with a reduction in ESRI, but rather appears to be a consequence of how the configuration space is explored. Indeed, “configuration model” simulations (unbiased rewiring at $\beta =0$), significantly increase *L* without changing $\langle \text {ESRI}\rangle$, thus showing that increasing *L* is not a sufficient condition for risk mitigation. In SI section S6, we show in detail the trajectories of *L*. Another evident pattern is that reciprocity is substantially reduced in the risk-mitigated network. Reciprocity is overexpressed in real production networks with respect to a suitably defined configuration model^17^, and it is clear that our rewiring algorithm, which does not constrain reciprocity levels, will necessarily lower them. Simulations with $\beta =0$ confirm this result, see SI section S6. We find that rewiring the unweighted networks reduces the size of the largest strongly connected components (SCC) in the Hungarian networks but not in the Ecuadorian ones. Interestingly, the largest SCCs in the Hungarian networks are small (less than 21%). Given that we do not observe a similar shrinking of the SCC for the other networks, this can be ruled out as a driving factor for systemic risk mitigation. In general, we could not find a pattern between systemic risk mitigation and any change in network measures, such as clustering, diameter, or average path length. In SI section S8, we check whether an increase in a firm’s degree leads to higher ESRI, as suggested by their correlation, or instead, reduces it due to the diversification effect.

The lower risk mitigation potential in the Hungarian sub-SCNs may result from the two different ways of isolating the subnetworks. While for Ecuador we first select firms in a target sector and then add all firms in the first tier of suppliers and customers, in the case of Hungary we used a clustering algorithm to identify communities of strongly interconnected nodes; see SI section S1. It is clear that risk reduction depends on the properties of the subnetwork. In this case, the lower mitigation potential might be associated with the lower average degree in the Hungarian networks as well as the considerably smaller size of the largest SCC (see Table 1). Except for these properties, we find no particular network measure that is directly associated with systemic risk mitigation. This suggests that systemic risk depends on the network structure at a mesoscale, as explored in^21^, which is not captured by the commonly used measures.

We further investigate the type of links in the risk-mitigated networks. We find that 35-55% of the original links are kept in the mitigated network, 5–8% of the links in the rewired networks were not present in the empirical snapshot of that year but appeared in other years, and 40–55% of links were never observed in the data. For each subnetwork, these counts are compatible with random rewiring as measured by the configuration model run of our simulation and are comparable with empirical rates of year-on-year changes^27^.

**Table 1:** Risk mitigation potential and network properties of six supply chain networks, before and after rewiring. The first column shows the average ESRI values for each empirical, risk-mitigated, and configuration model (unbiased rewiring at $\beta =0$) network. The second column reports the risk reduction in %. To see which topological changes lead to the observed systemic risk mitigation, we compare several network measures before and after rewiring. For reference, we include the values of the configuration model ($\beta =0$), taking their average value in the random network exploration after the burn-in steps. Measures marked by $\star$ are computed in the respective undirected network, where multi-edges were collapsed to a single link. We find no particular topological measure that explains systemic risk mitigation. This supports the hypothesis that systemic risk emerges from meso-scale structures that cannot be captured by network local measures or global network metrics.

		$\langle \text {ESRI}\rangle$	$\langle \text {ESRI}\rangle$ reduction	N	L	$\langle k_{tot}\rangle$	$\langle \langle k_{tot}\rangle _{\text {NN}} \rangle$	Global clustering coefficient	Diameter$^\star$	Average shortest path$^\star$	Size of the 3 largest SCCs	Size of the largest WCC	Reciprocity
Weighted NWs (Ecuador)													
Crustaceans	Empirical	0.794E−02	–	1075	7128	13.26	15.15	2.6E−05	7	2.91	[736,2,2]	1075	12.0%
	Risk-mitigated	0.458E−02	− 42.4%	–	22265	41.42	33.29	0.7E−05	5	2.25	[760,2,1]	1075	7.4%
	Configuration model ($\beta =0$)	0.808E−02	+1.8%	–	21620.6	40.22	33.68	0.7E−05	5.05	2.26	[761,2,1]	1075.0	7.6%
Soft drinks	Empirical	1.975E−02	–	890	9323	20.95	45.16	2.3E−05	6	2.38	[545,2,2]	890	14.8%
	Risk-mitigated	0.988E−02	− 50.0%	–	13177	29.61	39.08	0.9E−05	5	2.26	[542,1,1]	890	8.7%
	Configuration model ($\beta =0$)	2.154E−02	+9.1%	–	13329.3	29.95	39.32	0.9E−05	5.10	2.27	[568,1,1]	890.0	9.2%
Unweighted NWs (Ecuador)													
Crustaceans	Empirical	0.804E−02	–	1075	7128	13.26	15.15	2.35E−01	7	2.91	[736,2,2]	1075	12.0%
	Risk-mitigated	0.452E−02	− 43.7%	–	–	–	16.65	1.51E−01	5	2.76	[737,2,1]	1075	7.7%
	Configuration model ($\beta =0$)	0.726E−02	− 9.7%	–	–	–	15.58	1.88E−01	6.1	2.76	[749,2,1]	1075.0	3.4%
Soft drinks	Empirical	1.142E−02	–	890	9323	20.95	45.16	3.08E−01	6	2.38	[545,2,2]	890	14.8%
	Risk-mitigated	0.933E−02	− 18.3%	–	–	–	49.36	2.95E−01	5	2.32	[542,2,1]	890	10.4%
	Configuration model ($\beta =0$)	1.220E−02	+6.8%	–	–	–	45.23	3.21E−01	5.48	2.34	[565,1,1]	889.7	10.4%
Unweighted NWs (Hungary)													
Food production	Empirical	0.455E−02	–	1062	2342	4.41	7.33	6.47E−02	9	3.91	[127,14,6]	1062	8.5%
	Risk-mitigated	0.381E−02	− 16.1%	–	–	–	7.55	5.63E−02	9	3.86	[62,6,5]	1062	5.6%
	Configuration model ($\beta =0$)	0.472E−02	+3.7%	–	–	–	7.37	5.13E−02	9.71	3.86	[194,5,3]	1054.6	2.8%
Automotive	Empirical	0.550E−02	–	1147	2561	4.47	4.37	8.97E−02	11	4.15	[237,3,3]	1147	12.8%
	Risk-mitigated	0.367E−02	− 33.3%	–	–	–	4.36	4.93E−02	10	4.05	[128,3,3]	1147	7.7%
	Configuration model ($\beta =0$)	0.590E−02	+7.3%	–	–	–	4.25	4.60E−02	10.17	4.05	[279,4,3]	1133.7	5.7%

## Discussion

Traditional approaches to increasing the resilience of supply chains typically adopt a firm-level perspective, emphasizing different strategies available to firms, including higher inventory levels or supplier redundancy^33^. Both strategies come at the expense of efficiency and can entail significant costs. While economic literature has largely focused on the debate on reshoring global value chains (GVCs)^34,35^ and maintaining strategic stockpiles for critical industries^36^, these measures do not fully address systemic risk in supply networks. Similarly, management science has a well-established tradition of examining resilience, primarily through the lens of the flexibility-versus-redundancy debate^37^. However, the role of network topology in mitigating supply chain disruptions has been largely overlooked. Here, we propose an alternative approach that leverages topological considerations to reduce systemic risk. Rewiring supply chain networks (SCNs) does not necessarily impose additional costs or compromise efficiency. Empirical studies indicate that firms adjust supply links at a surprisingly rapid pace^27^, suggesting that the associated costs may be relatively low for a majority of links. We focus on targeted restructuring of SCNs to limit the spread of local disruptions and prevent cascading failures across the broader economy, thereby mitigating costly supply chain crises. Our findings demonstrate that such rewiring can achieve up to a 50% reduction in systemic risk without diminishing firms’ production capacity.

In particular, we find a risk mitigation potential of -42% and -50% for the Ecuadorian “crustacean” and “soft drinks” production networks, respectively. Topological risk mitigation is even possible for unweighted networks which underscores the essential role of topology in economic systemic risk. We show that similar percentages can be reached in situations where only the topology of supply relations is known but transaction volumes are not. For this, we produce unweighted versions of the SCNs and find a risk mitigation potential of -44% and -19%, respectively. We demonstrate that the risk mitigation margins vary with the specific production network under study. We analyzed different production sectors in two countries, finding an overall range of topological risk reduction between -16% and -50%. The two countries considered differ in their levels of economic development and in the types of relevant industries. Despite these country-specific features, the subnetworks that correspond to various industries within their national supply-chain networks, show similar magnitudes of systemic risk mitigation potential. This suggests that the observed reduction potential is not driven primarily by national economic characteristics, but rather by structural features of production networks that are common across economies. Our results reinforce the existence of large margins of topological systemic risk reduction, aligning with the levels observed in various financial market contexts, such as interbank markets^28,30,38^, bank stress testing of overlapping portfolios^31^, and multi-layer exposures^26^.

To understand the mechanisms of topological systemic risk mitigation, we correlate the risk reduction with the corresponding changes in a series of local network measures, such as degree, clustering, reciprocity, and global parameters, such as diameter and size of strongly and weakly connected components. We find no conclusive evidence that a network measure alone could explain the risk reduction. We relate this to the existence of a “systemic risk core” in SCNs, as reported in^21^. Such a core consists of those firms that carry the bulk of the systemic risk. The existence of a core is indicated by a plateau of high systemic risk firms in the systemic risk profile, as can be seen in the “Empirical NW” profiles in Fig. 4. Many of the firms within the core are mutually connected, and the failure of one of them typically leads to the failure of most firms in the core. This also explains the formation of the plateau, since any failure would lead to the same set of collapsing firms (creating the same total losses reflected in ESRI). The core is neither characterized by local network properties nor global ones; it is a network structure at a meso level. Any reconfiguration of the core has a large impact on the total systemic risk in the system. Since this meso-structure is not captured by traditional network measures, and it is not trivial to reduce it to a single value, it is reasonable that we cannot attribute the observed systemic risk reduction to one of the network measures alone.

The existence of a risk mitigation potential in the observed six examples means that actual production networks are suboptimal with respect to systemic risk. This is somewhat expected since firms do not and cannot consider systemic risk for decisions on how to choose their suppliers. We find that the levels of systemic risk observed in actual production networks are roughly the same as those that we get with random rewiring using the mentioned constraints. We reach lower levels only when we impose the risk reduction bias in the rewiring. This means that if one could make systemic risk information visible to firms, and if this risk could become a factor in their choice of suppliers through the internalization of this cost, market forces would bring the system toward lower systemic risk levels. We acknowledge that our data do not allow us to investigate the welfare costs of the proposed intervention in terms of loss of productivity or increases in consumer prices. Further, there are many considerations that go into the selection of suppliers, for example, the quality of their products^39^, which might further constrain or introduce costs to our proposed method. We have nevertheless established the potential effectiveness of this structural approach to risk mitigation and leave further analysis of the implications and cost-benefit analysis to future studies.

An obvious limitation of the constraints used to generate the space of allowed networks is that we do not have explicit information on the products the firms actually exchange. We proxy firms’ products by the NACE classifications of the selling firm, which can introduce errors in the computation of their systemic risk. When it comes to estimating the rewiring options of firms – the sets of potential suppliers for firms – instead we use as constraints both the target and source node industry classification. This further restriction should ensure that we are more likely to be underestimating the potential systemic risk reduction than overestimating it. Unfortunately, presently there is a lack of data on product information for firm-level networks, making the estimation of the sensitivity of our result to this classification a daunting task. Indeed, the error could go in both directions; the method could be too stringent, by limiting possible swaps to firms in sector pairs where we observe links, or it could be too lax, by assuming that firms in the same sector are mutually substitutable, where, in reality, they specialize in very different production. Nevertheless, the presence of supplier competition within industries provides empirical evidence of some degree of supplier substitutability, and indeed, this dynamics is captured by the rewiring rates from one year to another in the data. A further data limitation is that we do not have information on final demand, meaning that the size of every firm in the network could have been wrongly assessed. Only the availability of more detailed data will make it possible to improve these shortcomings.

In a similar direction, a further limitation due to the lack of product information arises with the definition of systemic risk itself. The computation of ESRI involves estimates of the “substitutability” of firms in the SCN and their production functions. The current approach involves an approximation and is known to be subject to further improvement until data with more detailed product information become available. These issues are well known and were discussed in the literature before^21^.

The presented rewiring algorithm could be improved by incorporating the heterogeneity of the rewiring costs. Supply links where the involved parties have invested heavily can be more difficult to replace, and this increases the systemic risk associated with them^40^. However, this is not necessarily reflected in long-term adjustments, and a supplier that is impossible to replace during a crisis could be replaced at a small cost over a few-year horizon. Although detailed firm-level data on supply-link-specific rewiring costs are not available to us, we can proxy these costs with the persistence of links over time. We find no indication that persistent links are preferentially untied and rewired in the risk-mitigated networks compared with simulated networks with the same systemic risk as the empirical networks (the configuration model).

Another immediate extension could be considering the geographic distance between companies and its influence on establishing supplier-buyer relations^41^. The preference of firms for ties to nearby businesses could be added as a further constraint to the rewiring algorithm or as a bias in the probability of accepting the swap. Doing so could help the model retain the well-documented tendency of firms to co-locate for the positive externalities this entails, especially in terms of labor pooling and knowledge transfers^42^.

A challenge that is intrinsic to the presented approach is the size of the “configuration space” of SCNs. The number of possible networks producing more or less the same set of intermediate and final products is immense. The chances that a rewiring procedure finds the globally optimal (minimal systemic risk) network configurations are practically zero. Therefore, here we do not search for the optimal network configurations, but we only make statements of how feasible it is to reach configurations that are significantly less risky. The situation is similar to many problems in statistical physics with large configuration spaces. There, the method of importance sampling has been applied with great success. Its strength is its ability to explore large configuration spaces effectively to find regions of low systemic risk. The problem of getting stuck in local minima can be ameliorated by superimposing methods like simulated annealing, which we successfully implemented here. In our simulations, we find that after several thousand rewiring iterations one converges towards relatively stable risk levels, with no guarantee of being close to the true optimum.

Depending on whether we use weighted or unweighted networks to reach approximate convergence one needs in the order of 100,000 and 10,000 rewiring steps, respectively. To find what these numbers mean in the context of actual SCN rewiring, one has to consider the number of links in the networks. This is approximately 10 steps per link in the weighted case (soft drinks), and less than one step per link in the unweighted case (food production). Interestingly, in the unweighted case, this roughly corresponds to the turnover of suppliers we would observe in 2-3 years^27^. In the weighted case currently our algorithm is not as efficient, however in both cases we are considering the steps necessary for the exploration of the configuration space rather than the minimal set of swaps necessary to reduce the systemic risk significantly. It is quite likely that a very small set of link changes is responsible for the majority of the mitigation we observe. Identifying these links, as well as the optimal steps necessary to achieve this reduction, is the subject of further study.

Given the potentially large economic cost associated with systemic risk arising from supply chain failures, several policy implications arise from this work. Although this work is focused on assessing systemic risk mitigation potential and does not explicitly model firm behavior, it identifies benchmark levels against which such mitigation can be compared. Considering the high level of turnover of supply relations in real supply chain networks, relatively small incentives or ’nudges’ for firms to consider alternative network configurations could, in principle, be sufficient to achieve significant systemic risk reduction. Such incentives could be similar to suggestions of topological risk mitigation in financial systems, such as systemic-risk insurance schemes^28,29^. Implementing comparable approaches in supply chain networks would require to regularly monitor systemic risk at the firm-level. At present, firm-level supply-chain data are available only in a limited number of countries, and if available, they are typically not accessible to firms. Designing mechanisms that provide incentives for supply-chain rewiring while preserving firm competitiveness and avoiding adverse effects, e.g., on consumer prices, remains an open challenge and is left here for future work.

## Materials and methods

### Economic systemic risk index

To estimate the systemic risk content of a given production network we use the firm average Economic Systemic Risk Index (ESRI)^21^. Each firm *i*, in the network is assumed to produce according to its generalized Leontief production function,

$$\begin{aligned} x_i= \min \left[ \min _{k\in \mathcal {I}_i^{\text {es}}}\left[ \frac{1}{\alpha _{ik}}\Pi _{ik} \right] , \bar{\beta }_i + \frac{1}{\alpha _i}\sum _{k\in \mathcal {I}_i^{\text {ne}}}\Pi _{ik}, \frac{1}{\alpha _{l_i}}l_i, \frac{1}{\alpha _{c_i}}c_i, \right] . \end{aligned}$$

where $\Pi _{ik}=\sum _j W_{ji}\delta _{p_j,k}$ is the amount of input *k* firm *i* uses for production, $\mathcal {I}_i^{\text {es}}$ and $\mathcal {I}_i^{\text {ne}}$ represent the set of essential and non-essential inputs of firm *i*, and $l_i$ and $c_i$ are *i*’s labor and capital inputs. Parameter $\bar{\beta }_i$ is the production level possible without non-essential inputs, and $\alpha$ is the matrix of technological coefficients. These parameters are calibrated on the empirically observed inputs of every firm. Every firm produces a single product described by its economic activity classification (NACE 3-digit). Whether a link constitutes an essential input or not is determined according to an expert-based survey for 56 sectors conducted by^6^. The survey identifies industries that provide inputs that other industries cannot easily substitute on short notice. The ESRI value of a firm is determined by setting the production level of the firm to zero and measuring the impact of this reduction both upstream and downstream of the firm. The resulting loss of total output is used to measure the systemic importance of the firm subject to the initial shock. ESRI$_i$ is the fraction of total production losses in the entire production network as a consequence of the failure of firm *i*. For more details, consult the SI in^21^.

### Supply chain networks

The six networks used in the analysis are derived from national VAT payments, representing the yearly aggregate taxes paid on firm-to-firm transactions in 2017 for Hungary and in 2015 for Ecuador. From these nationwide VAT graphs, we extract six subgraphs. Specifically, from the Ecuadorian network, we obtain two weighted graphs: one with 1,075 firms and 7,128 links (crustaceans) and another with 890 firms and 9,323 links (soft drinks), along with their unweighted counterparts. Similarly, from the Hungarian network, we derive two unweighted graphs: one with 1,062 firms and 2,342 links (food production) and another with 1,147 firms and 2,561 links (automotive). The subnetworks are extracted to analyze specific production subsystems. We employ two methods, one based on identifying clusters of nodes using a greedy modularity optimization for the Hungarian network, and one based on a set of seed nodes for which Tier-1 suppliers and customers from relevant industries were then added in the case of Ecuador. These strategies have been designed to guarantee that the smaller subsystems have sufficient redundancy, both in terms of firms with similar economic activities and in the links connecting them, to ensure that internal reorganization is possible. We refer to the SI section S1 for more details.

### Metropolis-Hastings algorithm and simulated annealing

A link rewiring step from a given network configuration, *a*, is proposed that would lead to a new configuration, *b*. The decision to accept or reject the proposed rewiring step depends on whether it brings the network to a state characterized by lower systemic risk. After evaluating the average value of the $\text {ESRI}$ in *b*, the probability of accepting the rewiring step is

1 $$\begin{aligned} p_{a\rightarrow b} = \min \{1, \exp (-\beta \,\Delta E_{ab})\} \quad , \end{aligned}$$

where

2 $$\begin{aligned} \Delta E_{ab} = \langle \text {ESRI}\rangle _b - \langle \text {ESRI}\rangle _a \quad . \end{aligned}$$

$\beta =1/T$ is sometimes called an “inverse temperature”, it is a parameter that controls the likelihood of accepting changes that increase the systemic risk, allowing the system to jump over local minima. We also employ a simulated annealing procedure, which often ameliorates the problem of getting stuck in local minima. To this end, we increase $\beta$ continuously with the number of iterations in the simulation.

## Supplementary Information

Supplementary Information.

## Data Availability

The datasets that support the findings of this study are the property of the Servicio de Rentas Internas (Ecuadorian VAT dataset) and the Magyar Nemzeti Bank, the central bank of Hungary (Hungarian VAT dataset). Access to these data is restricted under Non-Disclosure Agreements. Researchers seeking additional information may contact Pablo Astudillo-Estevez (Ecuador: pastudillo@usfq.edu.ec) and András Borsos (Hungary: borsosa@mnb.hu).

## References

[1] Craighead, C. W., Blackhurst, J., Rungtusanatham, M. J. & Handfield, R. B. The severity of supply chain disruptions: design characteristics and mitigation capabilities. *Decis. Sci.* **38**, 131–156 (2007).

[2] Choi, T. Y., Netland, T. H., Sanders, N., Sodhi, M. S. & Wagner, S. M. Just-in-time for supply chains in turbulent times. *Prod. Oper. Manag.* **32**, 2331–2340 (2023).

[3] Haraguchi, M. & Lall, U. Flood risks and impacts: A case study of Thailand’s floods in 2011 and research questions for supply chain decision making. *Int. J. Disaster Risk Reduction* **14**, 256–272 (2015).

[4] Carvalho, V. M., Nirei, M., Saito, Y. U. & Tahbaz-Salehi, A. Supply chain disruptions: Evidence from the Great East Japan earthquake*. *Q. J. Econ.* **136**, 1255–1321 (2020).

[5] Inoue, H. & Todo, Y. Firm-level propagation of shocks through supply-chain networks. *Nat. Sustain.* **2**, 841–847 (2019).

[6] Pichler, A., Pangallo, M., del Rio-Chanona, R. M., Lafond, F. & Farmer, J. D. Forecasting the propagation of pandemic shocks with a dynamic input-output model. *J. Econ. Dyn. Control* **144**, 104527 (2022). 36117523 10.1016/j.jedc.2022.104527 PMC9472492

[7] del Rio-Chanona, R. M., Mealy, P., Pichler, A., Lafond, F. & Farmer, J. D. Supply and demand shocks in the covid-19 pandemic: An industry and occupation perspective. *Oxf. Rev. Econ. Policy* **36**, S94–S137 (2020). 10.1093/oxrep/graa033 PMC7499761 40504218

[8] Bonadio, B., Huo, Z., Levchenko, A. A. & Pandalai-Nayar, N. Global supply chains in the pandemic. *J. Int. Econ.* **133**, 103534 (2021). 34866652 10.1016/j.jinteco.2021.103534 PMC8633421

[9] Ascari, G., Bonam, D. & Smadu, A. Global supply chain pressures, inflation, and implications for monetary policy. *J. Int. Money Financ.* **142**, 103029 (2024).

[10] Kent, P. & Haralambides, H. A perfect storm or an imperfect supply chain? The US supply chain crisis. *Maritime Econ. Logistics* **24**, 1–8 (2022).

[11] Ramani, V., Ghosh, D. & Sodhi, M. S. Understanding systemic disruption from the covid-19-induced semiconductor shortage for the auto industry. *Omega* **113**, 102720 (2022). 35966134 10.1016/j.omega.2022.102720 PMC9363154

[12] Ben Hassen, T. & El Bilali, H. Impacts of the Russia-Ukraine war on global food security: Towards more sustainable and resilient food systems?. *Foods* **11**, 2301 (2022). 35954068 10.3390/foods11152301 PMC9368568

[13] Laber, M., Klimek, P., Bruckner, M., Yang, L. & Thurner, S. Shock propagation from the Russia-Ukraine conflict on international multilayer food production network determines global food availability. *Nat. Food* **4**, 508–517 (2023). 37322302 10.1038/s43016-023-00771-4

[14] The White House. FACT SHEET: President Biden Takes Action to Protect American Workers and Businesses from China’s Unfair Trade Practices. https://www.commerce.gov/news/fact-sheets/2024/05/fact-sheet-president-biden-takes-action-protect-american-workers-and (2024).

[15] European Commission. Commission imposes definitive countervailing duties on imports of battery electric vehicles from China. https://ec.europa.eu/commission/presscorner/detail/en/ip_24_5589 (2024).

[16] Draghi, M. The Future of European Competitiveness - A Competitiveness Strategy for Europe (2024). https://commission.europa.eu/topics/strengthening-european-competitiveness/eu-competitiveness-looking-ahead_en.

[17] Lafond, F., Astudillo-Estévez, P., Bacilieri, A. & Borsos, A. Firm-level production networks: what do we (really) know? INET Oxford Working Papers 2023–08, Institute for New Economic Thinking at the Oxford Martin School, University of Oxford (2023). https://ideas.repec.org/p/amz/wpaper/2023-08.html.

[18] Pichler, A. et al. Building an alliance to map global supply networks. *Science* **382**, 270–272 (2023). 37856603 10.1126/science.adi7521

[19] Choi, T. Y. & Krause, D. R. The supply base and its complexity: Implications for transaction costs, risks, responsiveness, and innovation. *J. Oper. Manag.* **24**, 637–652 (2006).

[20] Diem, C., Borsos, A., Reisch, T., Kertész, J. & Thurner, S. Estimating the loss of economic predictability from aggregating firm-level production networks. PNAS Nexus 3, pgae064 (2024). 10.1093/pnasnexus/pgae064 PMC10965025 38533108

[21] Diem, C., Borsos, A., Reisch, T., Kertész, J. & Thurner, S. Quantifying firm-level economic systemic risk from nation-wide supply networks. *Sci. Rep.* **12**, 7719 (2022). 35546595 10.1038/s41598-022-11522-z PMC9092945

[22] Acemoglu, D. & Tahbaz-Salehi, A. The macroeconomics of supply chain disruptions. *Rev. Econ. Stud.* **92**, 656–695 (2024).

[23] Boss, M., Elsinger, H., Summer, M. & Thurner 4, S. Network topology of the interbank market. *Quant. Finance* 4, 677–684 (2004).

[24] Iori, G., Jafarey, S. & Padilla, F. G. Systemic risk on the interbank market. *J. Econ. Behav. Organ.* **61**, 525–542 (2006).

[25] Battiston, S., Puliga, M., Kaushik, R., Tasca, P. & Caldarelli, G. DebtRank: Too central to fail? Financial networks, the FED and systemic risk. *Sci. Rep.* **2**, 1–6 (2012). 10.1038/srep00541 PMC3412322 22870377

[26] Poledna, S., Molina-Borboa, J. L., Martínez-Jaramillo, S., van der Leij, M. & Thurner, S. The multi-layer network nature of systemic risk and its implications for the costs of financial crises. *J. Financ. Stab.* **20**, 70–81 (2015).

[27] Reisch, T., Borsos, A. & Thurner, S. Supply chain network rewiring dynamics at the firm-level. Preprint at arXiv:2503.20594 (2025). 10.1093/pnasnexus/pgag091 PMC13126663 42063671

[28] Poledna, S. & Thurner, S. Elimination of systemic risk in financial networks by means of a systemic risk transaction tax. *Quant. Finance* **16**, 1599–1613 (2016).

[29] Leduc, M. V. & Thurner, S. Incentivizing resilience in financial networks. *J. Econ. Dyn. Control* **82**, 44–66 (2017).

[30] Diem, C., Pichler, A. & Thurner, S. What is the minimal systemic risk in financial exposure networks?. *J. Econ. Dyn. Control* **116**, 103900 (2020).

[31] Pichler, A., Poledna, S. & Thurner, S. Systemic risk-efficient asset allocations: Minimization of systemic risk as a network optimization problem. *J. Financ. Stab.* **52**, 100809 (2021).

[32] Regulation (EC) No 1893/2006 of the European Parliament and of the Council of 20 December 2006 establishing the statistical classification of economic activities NACE Revision 2 and amending Council Regulation (EEC) No 3037/90 as well as certain EC Regulations on specific statistical domains Text with EEA relevance (2006).

[33] Ho, W., Zheng, T., Yildiz, H. & Talluri, S. Supply chain risk management: A literature review. *Int. J. Prod. Res.* **53**, 5031–5069 (2015).

[34] Baldwin, R. & Freeman, R. Risks and global supply chains: What we know and what we need to know. *Annu. Rev. Econ.* **14**, 153–180 (2022).

[35] König, M. D., Levchenko, A., Rogers, T. & Zilibotti, F. Aggregate fluctuations in adaptive production networks. *Proc. Natl. Acad. Sci.* **119**, e2203730119 (2022). 36095207 10.1073/pnas.2203730119 PMC9499558

[36] Sprecher, B. et al. Framework for resilience in material supply chains, with a case study from the 2010 rare earth crisis. *Environ. Sci. Technol.* **49**, 6740–6750 (2015). 25965803 10.1021/acs.est.5b00206

[37] Kamalahmadi, M. & Parast, M. M. A review of the literature on the principles of enterprise and supply chain resilience: Major findings and directions for future research. *Int. J. Prod. Econ.* **171**, 116–133 (2016).

[38] Thurner, S. & Poledna, S. Debtrank-transparency: Controlling systemic risk in financial networks. *Sci. Rep.* **3**, 1888 (2013). 23712454 10.1038/srep01888 PMC3664900

[39] Demir, B., Fieler, A. C., Xu, D. Y. & Yang, K. K. O-ring production networks. *J. Polit. Econ.* **132**, 200–247 (2024).

[40] Barrot, J.-N. & Sauvagnat, J. Input specificity and the propagation of idiosyncratic shocks in production networks *. *Q. J. Econ.* **131**, 1543–1592 (2016).

[41] Bernard, A. B., Moxnes, A. & Saito, Y. U. Production networks, geography, and firm performance. *J. Polit. Econ.* **127**, 639–688 (2019).

[42] Juhász, S., Elekes, Z., Ilyés, V. & Neffke, F. Colocation of skill related suppliers-revisiting coagglomeration using firm-to-firm network data. Preprint at arXiv:2405.07071 (2024).

