# Supplementary material for: Systemic risk mitigation in supply chains through network rewiring
Source: Sci Rep. 2026 Mar 5;16:12334. doi: 10.1038/s41598-026-42549-1 (PMC13079837; doi:10.1038/s41598-026-42549-1)
Supplement: Supplementary file 1 — Supplementary Information. [file 41598_2026_42549_MOESM1_ESM.pdf]

Supplementary Information for:
Systemic risk mitigation in supply chains through network rewiring

Giacomo Zelbi, Leonardo Niccolò Ialongo, and Stefan Thurner*

*Corresponding author, e-mail: stefan.thurner@meduniwien.ac.at

S1. DERIVATION OF THE SIX PRODUCTION NETWORKS

Our analysis is based on nationwide supply chain network (SCN) data from Ecuador and Hungary. These datasets are derived from value-added tax (VAT) payment records, capturing firm-to-firm transactions, with each direction of payment between firms (i.e., $i \to j$ and $j \to i$) recorded as a separate entry. Firms are anonymized in both datasets, but their economic activity classifications are available - ISIC codes [1] for Ecuador and NACE codes [2] for Hungary. The transactions are aggregated annually, and data are available for multiple years. For Ecuador we have access to the transactions' aggregated volume, while for Hungary only the information about the existence of the transactions is known. Because the size of the nationwide SCNs is too large to simulate the disruption cascade of each node failure at every Monte Carlo rewiring step, we derive two weighted subnetworks from the Ecuadorian data (2015), their two unweighted counterparts, and two unweighted subnetworks from the Hungarian data (2017).

A. Extracting two (plus two) production networks from the countrywide Ecuadorian SCN

The Ecuadorian dataset consists of VAT payments from 2015, capturing ~ 14 million yearly-aggregated transactions between ~ 1 million distinct anonymized taxpayers. Each row represents the total monetary flow from one taxpayer to another. To refine the data, we excluded transactions involving less than $3,000$ USD, self-loops (transactions where the payer and payee are the same), and entries involving entities labeled as *not defined*, *no economic activity*, or requiring *verification*. Furthermore, we removed entries belonging to the *personas naturales* type of tax contributor. After these filters, the dataset was reduced to almost 650 thousand transactions between ~ 65 thousand taxpayers. The cleaned data were then used to construct a weighted directed SCN where the direction of the links is opposite to the flow of money, and the link weight is interpreted as the volume of goods exchanged. In this network, a firm's in-neighbors (out-neighbors) represent its suppliers (customers). The firms are originally categorized according to their economic activity in the ISIC scheme, which we converted to NACE.

The crustaceans network was derived from the 65k-firms network as follows. We notice that Ecuador's 2015 export basket (atlas.cid.harvard.edu) from the Atlas of Economic Complexity [3] identifies "Crustaceans" and "Prepared or preserved fish" as contributing approximately 12.35% of the country's gross exports. This significant share suggests that the national production network is rich and developed enough to try to capture it through the extraction of a subgraph. First, within the 65k firms filtered SCN, we selected the 109 firms belonging to NACE 4-digit *class* C1020 ("Processing and preserving of fish, crustaceans, and molluscs"). Second, we considered the set of all their in-neighbors (suppliers), excluding those also classified under C1020. The NACE 3-digit *groups* of this set of firms were ranked based on their overrepresentation relative to their distribution in the 65-k firms SCN. From this ranking, we selected the top sixteen groups, ensuring that each contained at least five firms. This minimum threshold helped mitigate small-network effects, where having only a few representatives of a given NACE group in the subnetwork could artificially inflate their systemic importance due to their low replaceability within the subset. Third, we applied the same procedure to the out-neighbors (customers) of the C1020 firms, selecting the eight most overrepresented NACE groups. Fourth, we induced a subnetwork from the larger 65k-firms SCN, comprising the C1020 firms, their Tier 1 suppliers and customers from the selected groups, and all the links between these nodes. Finally, we considered the largest weakly connected component of this subnetwork. This approach, selecting overrepresented groups, was intentionally tailored to yield a network of manageable size while emphasizing economic activities that characterize the companies in the NACE class C1020 and distinguish this production network from the broader national supply chain network.

The derivation of the soft drinks network followed the same procedure, focusing on different economic activities. Here, we targeted the nodes in NACE 4-digit class C1107 (Manufacture of soft drinks; production of mineral waters and other bottled waters), whose output should represent a final product requiring no further processing. The NACE 3-digit groups of Tier 1 suppliers and customers for firms in C1107 exhibited greater diversity, reflecting the complexity expected for highly processed products. For the induced subgraph, we selected nodes from the top 23 overrepresented supplying NACE groups and the top 20 buying groups. Note that the links in these two subnetworks are weighted. Deriving the unweighted version of the two networks is trivial, we removed the information on the link weights simplifying their values to one.

B. Extracting two production networks from the countrywide Hungarian SCN

The Hungarian dataset is also derived from payments between companies based in the country to the tax authorities, reporting supply invoices for VAT purposes. The identity of the firms is anonymized and not available to us. The data are available for every year, starting from 2015. Here we considered 2017, and the transactions are aggregated for the entire year. In 2017, only payments with tax content exceeding the threshold of 1 million HUF ($\sim$ 3500 USD, in that year) were required to be reported. In this work we do not have access to the link weights, and this means that the directed links in the resulting network capture only the existence of supply links, between companies exchanging goods or services with tax value above the threshold. As in the Ecuadorian case, the size of the countrywide network is too large to evaluate $\langle \text{ESRI} \rangle_f - \langle \text{ESRI} \rangle_i$ at every Monte Carlo step, so we extract two subnetworks in the following way. We first note that firms in manufacturing (NACE C) are very heterogeneous in the inputs that are essential to them for production (Fig.9 in [4]). Therefore the subnetwork of manufacturing firms already displays many of the properties of the larger network in terms of the complexity of the dependency structure. Hence, we isolate all the Hungarian firms in NACE section C and consider only the links between these firms. The induced subnetwork, is partitioned in communities with a Clauset-Newman-Moore greedy modularity maximization. After this step, we chose two communities with approximately 1000 nodes, and we looked at their composition in terms of NACE sectors. In Tab S1, we break down the composition of the communities in terms of NACE 3-digit groups. The names for the two communities, "food production" network and "automotive" network, were chosen after the most overrepresented sectors in the subnetworks compared to the original countrywide NW.

C. Properties of the networks

The distributions of NACE economic activities for the derived production networks are presented in Table S1. The rows break down the composition of each subnetwork, reporting the absolute number of firms categorized in each NACE group and the equivalent fraction of the network (in percentage). The most populated NACE groups are bolded for each network.

In Figure S1, we reported the Complementary Cumulative Distribution Function (CCDF) for several node and link attributes on a log-log scale. In Figure S1A, we plotted the CCDF of the node total degrees k^{tot}. The different colored lines correspond to the different networks. A clear pattern is visible, with the crustaceans and soft drinks lines positioned further to the right compared to the food production and automotive lines. This shift reflects the fact that nodes in these networks tend to have higher degrees. The maximum total degrees observed in the food production, automotive, crustaceans, and soft drinks networks are 195, 109, 305, and 527, respectively. By construction, the rewiring algorithm in the case of unweighted networks preserves in-degrees, out-degrees, and total degrees, so the plot does not change after the risk mitigation. Figure S1B illustrates the CCDF of link weights for the weighted Ecuadorian subnetworks, and compares them with the CCDF in the original nationwide SCN (Ecuador NW in the legend). As shown by the maximum x-axis (weight) values of the three lines, both induced subgraphs include links from the tail of the main distribution, overlapping with the higher-weight region of the Ecuador NW curve. The threshold on link weights applied before the subnetwork derivation introduces a truncation effect along the x-axis, preventing links with weights lower than $3,000$ USD from appearing in the induced graphs. Figure S1C presents the CCDF of node total strength s^{tot} for the same weighted networks. Here, the truncation on link weights affects node strengths reducing their overall values and preventing the existence of nodes with strengths below $3,000$.

NACE 3-digit groups	Food production	Automotive	Crustaceans	Soft drinks
031 Fishing	-	-	**98 (9.1%)**	8 (0.9%)
032 Aquaculture	-	-	**422 (39.3%)**	-
101 Processing and preserving of meat and production o	**68 (6.4%)**	-	-	-
102 Processing and preserving of fish, crustaceans and	-	-	**97 (9.0%)**	13 (1.5%)
103 Processing and preserving of fruit and vegetables	41 (3.9%)	-	-	6 (0.7%)
104 Manuf. of vegetable and animal oils and fats	4 (0.4%)	-	-	-
105 Manuf. of dairy products	25 (2.4%)	-	-	9 (1.0%)
106 Manuf. of grain mill products, starches and starch	22 (2.1%)	-	-	-
107 Manuf. of bakery and farinaceous products	**103 (9.7%)**	1 (0.1%)	-	7 (0.8%)
108 Manuf. of other food products	47 (4.4%)	-	-	22 (2.5%)
109 Manuf. of prepared animal feeds	48 (4.5%)	-	12 (1.1%)	-
110 Manuf. of beverages	**102 (9.6%)**	3 (0.3%)	29 (2.7%)	**63 (7.1%)**
120 Manuf. of tobacco products	1 (0.1%)	-	-	-
133 Finishing of textiles	1 (0.1%)	-	-	-
139 Manuf. of other textiles	8 (0.8%)	8 (0.7%)	-	-
141 Manuf. of wearing apparel, except fur apparel	5 (0.5%)	4 (0.3%)	-	-
142 Manuf. of articles of fur	1 (0.1%)	-	-	-
143 Manuf. of knitted and crocheted apparel	1 (0.1%)	-	-	-
152 Manuf. of footwear	-	1 (0.1%)	-	-
161 Sawmilling and planing of wood	15 (1.4%)	12 (1.0%)	-	-
162 Manuf. of products of wood, cork, straw and plaiti	28 (2.6%)	12 (1.0%)	-	-
171 Manuf. of pulp, paper and paperboard	5 (0.5%)	-	7 (0.7%)	-
172 Manuf. of articles of paper and paperboard	**62 (5.8%)**	16 (1.4%)	10 (0.9%)	10 (1.1%)
181 Printing and service activities related to printin	51 (4.8%)	15 (1.3%)	-	22 (2.5%)
182 Reproduction of recorded media	1 (0.1%)	1 (0.1%)	-	-
192 Manuf. of refined petroleum products	2 (0.2%)	-	-	-
201 Manuf. of basic chemicals, fertilisers and nitroge	15 (1.4%)	2 (0.2%)	-	31 (3.5%)
202 Manuf. of pesticides and other agrochemical produc	5 (0.5%)	-	-	-
203 Manuf. of paints, varnishes and similar coatings,	11 (1.0%)	2 (0.2%)	-	-
204 Manuf. of soap and detergents, cleaning and polish	11 (1.0%)	-	-	-
205 Manuf. of other chemical products	14 (1.3%)	2 (0.2%)	22 (2.0%)	16 (1.8%)
206 Manuf. of man-made fibres	1 (0.1%)	-	-	-
212 Manuf. of pharmaceutical preparations	-	2 (0.2%)	-	-
221 Manuf. of rubber products	2 (0.2%)	26 (2.3%)	-	-
222 Manuf. of plastic products	**136 (12.8%)**	**77 (6.7%)**	28 (2.6%)	44 (4.9%)
231 Manuf. of glass and glass products	1 (0.1%)	1 (0.1%)	-	-
232 Manuf. of refractory products	-	2 (0.2%)	-	-
233 Manuf. of clay building materials	2 (0.2%)	-	-	-
234 Manuf. of other porcelain and ceramic products	-	1 (0.1%)	-	-
235 Manuf. of cement, lime and plaster	2 (0.2%)	-	-	-
236 Manuf. of articles of concrete, cement and plaster	14 (1.3%)	2 (0.2%)	-	-
237 Cutting, shaping and finishing of stone	2 (0.2%)	-	-	-
239 Manuf. of abrasive products and non-metallic miner	8 (0.8%)	1 (0.1%)	-	-
241 Manuf. of basic iron and steel and of ferro-alloys	-	1 (0.1%)	-	-
243 Manuf. of other products of first processing of st	1 (0.1%)	2 (0.2%)	-	-
244 Manuf. of basic precious and other non-ferrous met	-	7 (0.6%)	-	-
245 Casting of metals	-	29 (2.5%)	-	-
251 Manuf. of structural metal products	23 (2.2%)	**76 (6.6%)**	-	-
252 Manuf. of tanks, reservoirs and containers of meta	2 (0.2%)	4 (0.3%)	-	-
253 Manuf. of steam generators, except central heating	1 (0.1%)	-	-	-
255 Forging, pressing, stamping and roll-forming of me	-	17 (1.5%)	-	-
256 Treatment and coating of metals; machining	26 (2.4%)	**247 (21.5%)**	-	-
257 Manuf. of cutlery, tools and general hardware	3 (0.3%)	**76 (6.6%)**	-	-
259 Manuf. of other fabricated metal products	10 (0.9%)	27 (2.4%)	-	-
261 Manuf. of electronic components and boards	3 (0.3%)	27 (2.4%)	-	-
262 Manuf. of computers and peripheral equipment	-	2 (0.2%)	-	-
263 Manuf. of communication equipment	3 (0.3%)	7 (0.6%)	-	-
265 Manuf. of instruments and appliances for measuring	6 (0.6%)	17 (1.5%)	5 (0.5%)	-
266 Manuf. of irradiation, electromedical and electrot	-	1 (0.1%)	-	-
267 Manuf. of optical instruments and photographic equ	-	2 (0.2%)	-	-

271 Manuf. of electric motors, generators, transformer	3 (0.3%)	11 (1.0%)	-	-
273 Manuf. of wiring and wiring devices	3 (0.3%)	9 (0.8%)	-	-
274 Manuf. of electric lighting equipment	2 (0.2%)	6 (0.5%)	-	-
275 Manuf. of domestic appliances	-	3 (0.3%)	-	-
279 Manuf. of other electrical equipment	2 (0.2%)	9 (0.8%)	-	-
281 Manuf. of general-purpose machinery	8 (0.8%)	24 (2.1%)	7 (0.7%)	-
282 Manuf. of other general-purpose machinery	22 (2.1%)	55 (4.8%)	-	16 (1.8%)
283 Manuf. of agricultural and forestry machinery	2 (0.2%)	5 (0.4%)	-	-
284 Manuf. of metal forming machinery and machine tool	-	20 (1.7%)	-	-
289 Manuf. of other special-purpose machinery	21 (2.0%)	50 (4.4%)	6 (0.6%)	-
291 Manuf. of motor vehicles	1 (0.1%)	5 (0.4%)	-	-
292 Manuf. of bodies (coachwork) for motor vehicles; m	-	3 (0.3%)	-	-
293 Manuf. of parts and accessories for motor vehicles	-	**77 (6.7%)**	-	-
301 Building of ships and boats	-	-	12 (1.1%)	-
302 Manuf. of railway locomotives and rolling stock	2 (0.2%)	4 (0.3%)	-	-
303 Manuf. of air and spacecraft and related machinery	-	1 (0.1%)	-	-
309 Manuf. of transport equipment n.e.c.	1 (0.1%)	-	-	-
310 Manuf. of furniture	1 (0.1%)	43 (3.7%)	-	-
323 Manuf. of sports goods	-	1 (0.1%)	-	-
324 Manuf. of games and toys	1 (0.1%)	2 (0.2%)	-	-
325 Manuf. of medical and dental instruments and suppl	1 (0.1%)	5 (0.4%)	-	-
329 Manufacturing n.e.c.	5 (0.5%)	4 (0.3%)	-	-
331 Repair of fabricated metal products, machinery and	31 (2.9%)	52 (4.5%)	20 (1.9%)	-
332 Installation of industrial machinery and equipment	13 (1.2%)	25 (2.2%)	-	-
351 Electric power generation, transmission and distri	-	-	-	10 (1.1%)
360 Water collection, treatment and supply	-	-	-	5 (0.6%)
370 Sewerage	-	-	-	6 (0.7%)
451 Sale of motor vehicles	-	-	-	20 (2.2%)
463 Wholesale of food, beverages and tobacco	-	-	**84 (7.8%)**	**73 (8.2%)**
466 Wholesale of other machinery, equipment and suppli	-	-	-	**71 (8.0%)**
469 Non-specialised wholesale trade	-	-	-	42 (4.7%)
471 Retail sale in non-specialised stores	-	-	-	**51 (5.7%)**
472 Retail sale of food, beverages and tobacco in spec	-	-	9 (0.8%)	13 (1.5%)
473 Retail sale of automotive fuel in specialised stor	-	-	-	26 (2.9%)
477 Retail sale of other goods in specialised stores	-	-	-	14 (1.6%)
502 Sea and coastal freight water transport	-	-	21 (2.0%)	-
512 Freight air transport and space transport	-	-	9 (0.8%)	-
521 Warehousing and storage	-	-	-	7 (0.8%)
522 Support activities for transportation	-	-	**149 (13.9%)**	-
552 Holiday and other short-stay accommodation	-	-	-	47 (5.3%)
561 Restaurants and mobile food service activities	-	-	-	**73 (8.2%)**
562 Event catering and other food service activities	-	-	10 (0.9%)	12 (1.3%)
563 Beverage serving activities	-	-	11 (1.0%)	13 (1.5%)
601 Radio broadcasting	-	-	-	7 (0.8%)
651 Insurance	-	-	-	12 (1.3%)
712 Technical testing and analysis	-	-	7 (0.7%)	-
731 Advertising	-	-	-	46 (5.2%)
732 Market research and public opinion polling	-	-	-	6 (0.7%)
781 Activities of employment placement agencies	-	-	-	5 (0.6%)
852 Primary education	-	-	-	9 (1.0%)
854 Higher education	-	-	-	5 (0.6%)
931 Sports activities	-	-	-	50 (5.6%)

TABLE S1: Cross-sectoral distribution of firms by NACE 3-digit groups across the networks (food production, automotive, crustaceans, and soft drinks). Percentages in parentheses represent the share of nodes within the given NACE category. For each network, bolded values highlight the most populated categories.

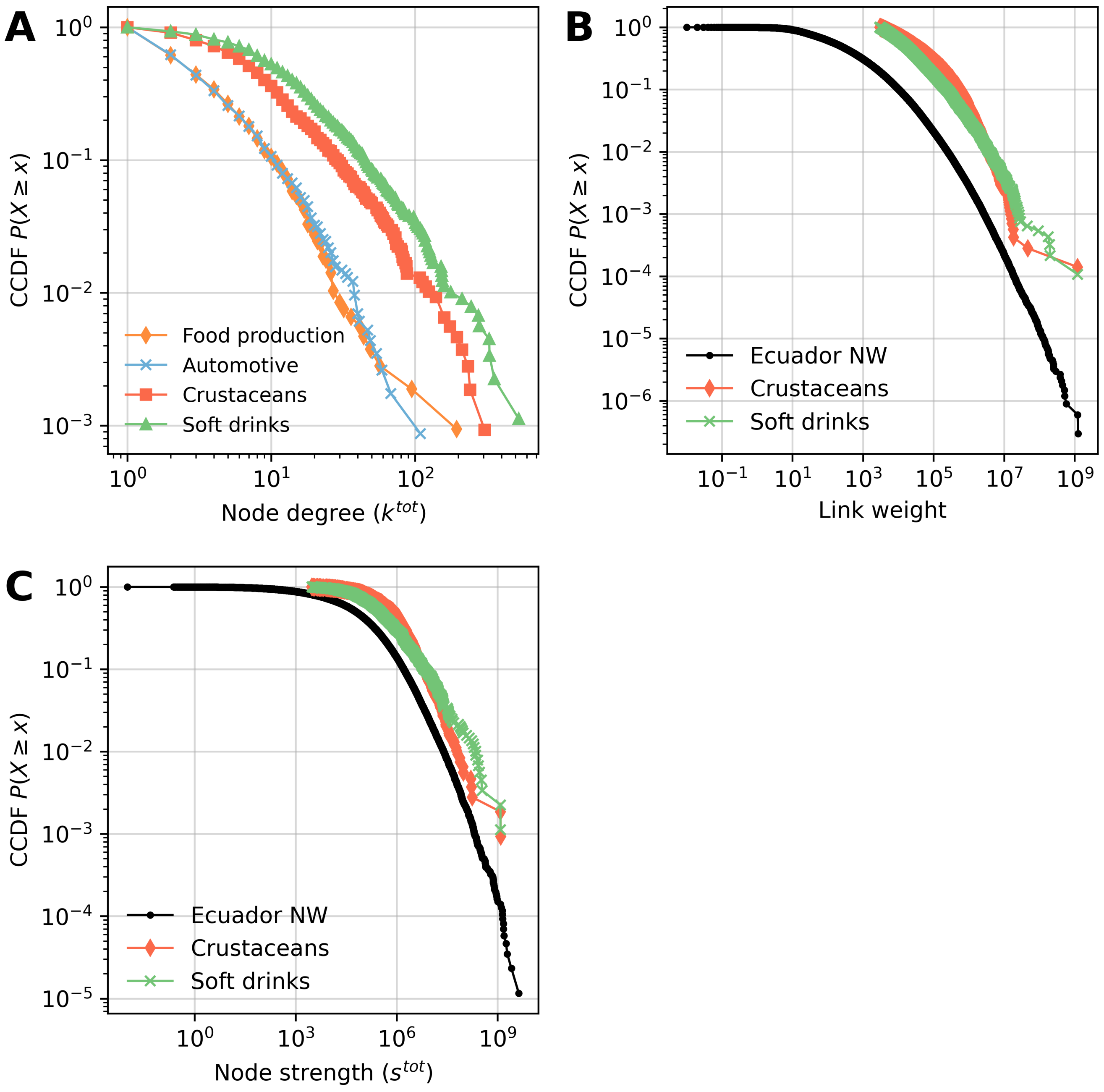

FIG. S1. Complementary cumulative distribution functions (CCDFs) for several network properties. Panel **A** reports the CCDFs of node total degree (k^{tot}) for the crustaceans, soft drinks, food production, and automotive networks. Node degrees do not change between the weighted and unweighted versions of the networks. In panel **B**, CCDF of node total strength (s^{tot}) comparing the Ecuadorian weighted subnetworks (crustaceans, soft drinks), and the original nationwide one (Ecuador network). In panel **C**, CCDF of link weight for the same weighted networks.

S2. ECONOMIC SYSTEMIC RISK INDEX (ESRI)

The Economic Systemic Risk Index (ESRI) quantifies a firm's systemic importance within a supply chain network by measuring the potential cascade of production failures triggered when the firm suddenly ceases operations. In the networks, each firm is represented as a node that produces goods or services ("products") using the entirety of the inputs sourced from suppliers (in-neighbors) and selling outputs to customers (out-neighbors). In- and out-neighbors are determined by the direction of the links, which follow the flow of products, opposite to the direction of the payments in the dataset. Since product-level information is unavailable, we use the NACE 3-digit classification of each firm as a proxy for the products it produces. This implies that firms within the same NACE group can supply the same products. The weight of the link from supplier j to customer i, denoted W_{ji}, represents the monetary value of the supplied product, serving as an estimate of the amount of product. Every firm is equipped with a generalized Leontief production function (GLPF), that models how the intermediate input quantities are transformed into output while distinguishing between essential and non-essential inputs. We employed the results of the survey conducted for [4] that classifies which NACE 2-digit division inputs are essential, non-essential, or irrelevant for production in each division. The possible production in each firm given input volumes and types is:

$$x_i = \min\left[\min_{k\in\mathcal{I}_i^{\text{es}}}\left[\frac{1}{\alpha_{ik}}\Pi_{ik}\right], \bar{\beta}_i + \frac{1}{\alpha_i}\sum_{k\in\mathcal{I}_i^{\text{ne}}}\Pi_{ik}, \frac{1}{\alpha_{l_i}}l_i, \frac{1}{\alpha_{c_i}}c_i\right]. \tag{1}$$

where $\Pi_{ik} = \sum_j W_{ji}\delta_{p_j,k}$ is the amount of input k firm i uses for production, $\mathcal{I}_i^{\text{es}}$ and $\mathcal{I}_i^{\text{ne}}$ represent the set of essential and non-essential inputs of firm i, and l_i and c_i are i's labor and capital inputs, which are untouched and ignored in our work settings. Essential inputs are treated in a Leontif way, and non-essential inputs affect production linearly. Parameter $\bar{\beta}_i$ is the production level possible without non-essential inputs, and α is the matrix of technological coefficients. This formulation captures firms' heterogeneity in input usage and substitutability.

To calculate ESRI of a firm j, we initialize the network by calibrating production functions at time $t = 0$. The production output that each firm i is able to sustain with the inputs delivered from the suppliers (calculated with (1)) is exactly equal to the summed volume of its sales transactions, the node outstrength s_i^{out}. Every firm i operates at full capacity, measured as $h_i(t = 0) = 1$. At $t = 1$, the exogenous shock hits and firm j ceases operations. This triggers both an upstream shock to suppliers and a downstream shock to customers. Firm j's suppliers face reduced demand and adjust their production level $h(t = 1)$, decreasing their output by the fraction that was supplied to j. Firm j's customers decrease production depending on the essentiality of the missing input in their production function. Essential inputs propagate shocks in a non-linear (Leontief) way, while non-essential inputs do so linearly. The impact of the lack of input in the production function is softened depending on how easy is to replace firm j as a supplier. The market share is used as a proxy for this replaceability: firms with higher market share are assumed to be harder to replace.

The shock propagates iteratively upstream and downstream, distributing the shock proportionally between the neighbors until convergence at time T. At T, the firms have decreased their empirical production level s^{out} by a factor $1 - h(T)$, and the ESRI of firm j is defined as the total production loss in the network:

$$\text{ESRI}_j = \sum_{i=1}^{n}\frac{s_i^{out}}{\sum_{l=1}^{n}s_l^{out}}\left(1 - h_i(T)\right) \quad . \tag{2}$$

Since rewiring is not permitted during cascade propagation, ESRI does not reflect dynamic adaptation over time. However, it is not designed to simulate real-world shock propagation in full detail, but rather to measure and provide insights into systemic relevance and the network's potential susceptibility to firm-level disruptions.

S3. DETAILS OF THE REWIRING ALGORITHM

A. Firms' production constraints require considering multiple links

We developed a Monte Carlo link-swapping algorithm to rewire weighted and unweighted networks while maintaining specific constraints. This method ensures that some supply chain networks' properties and firms' production capabilities remain consistent throughout the exploration of alternative configurations. In this paragraph, we provide a detailed explanation of the algorithm and its constraints, along with the extensions required to accommodate weighted networks.

Rewiring the network does not aim to model the temporal evolution of the firms forming it, but rather explore alternative configurations. For this reason, when rewiring a link, we aim to replace a supplier (customer) with another *equivalent* firm providing (demanding) the same specific product or service. In practice, however, detailed product-level information is often unavailable. Instead, firms are usually classified according to broad industrial schemes such as NACE or ISIC using different levels of granularity. In this case, we use the economic activity classification of each firm pair forming an edge as a proxy for the specific good being exchanged. The level of aggregation we chose is the NACE 3-digit group. Thus, we assume that firms in the same NACE group supplying from firms in another specific NACE group can freely choose to change suppliers from this second set. Of course, the algorithm is perfectly suited to work with more detailed product information. Rewiring should not alter the technology and productivity of the firms and then it must preserve the observed ratios and volumes of intermediate goods used to produce the levels of output. Because of the strong correlation between a node's connectivity and its systemic importance, we should also not alter the number of suppliers and customers of a firm. Finally, if a firm is empirically observed to purchase from a given number of suppliers providing the same intermediate good, we do not want to change this level of supply redundancy, which is a property of the firm. Similarly, the set of sectors a firm sells to and the number of customers in each sector should remain unchanged.

From a network perspective, these constraints amount to preserving both the in and out degree and strength per sector of each node. For simplicity consider first an unweighted network where each link has the same value of one unit. In this case, the degree and strength constraints are equivalent. These rules require considering more than one link in each rewiring step because deleting, introducing, or cutting and redirecting one single link would inevitably change the number of connections of its two old or new ends. The problem can be solved by rewiring multiple links rather than rewiring them individually. For simplicity, we considered two links per rewiring event. After selecting two links representing the same product - in our case with the same combination of (source-sector, target-sector), the suppliers of the two target nodes are exchanged. The same swap has a complementary point of view, in which the customers of the two source nodes are exchanged. For the illustration of the swap-rewiring, refer to Fig.2 of the main text. The swap is reversible, and this is important in the exploration of the configuration space as it guarantees that we can explore all possible network states without getting stuck.

This simple swap-rewiring has limitations: while degree and strength are strongly correlated, there can be significant heterogeneity of link weights. As such the simple swap discussed here does not preserve the production level of firms as strictly as we would like. However, we can alter the algorithm in such a way as to be more flexible on the degrees in exchange for tighter bounds on the strengths. To do so, we keep the same algorithm for the initial proposal of the swap. If the weights of two selected links differ, we look at the size of their difference: if the difference is sufficiently small, we deem the edges equivalent and perform the swap as usual. We considered sufficiently small a value below the threshold for the links in the original Ecuadorian supply chain network, 3000 USD. Note that to preserve the total weight of the graph, when performing the swap we will ensure that the in-strengths of the nodes are exactly kept, while the out-strengths can change slightly. This can be seen as anchoring the weight of the edge from the customer side and not from the supplier side. In production words, this means that the input levels of firms cannot change, while the output can. To ensure that the algorithm does not significantly change the output coefficients and production level by small increments or reductions, the node out-strengths are allowed to deviate by no more than 20% from their empirically observed values to accommodate this swap. In subsection D, we explore the dependence on this parameter. If the move would result in a violation of this strength constraint it is rejected. The other case to consider is if the difference between the two weights is too large to just perform a simple swap. In this case, we must choose between not performing the swap at all or violating the degree constraint by introducing a new link. We choose the latter, as this ensures more configurations can be explored given the small number of links that are exactly of the same value. Note that also these swap moves are all reversible, and as such, we can freely explore if the addition or subtraction of this link increases or decreases the systemic risk level of the network.

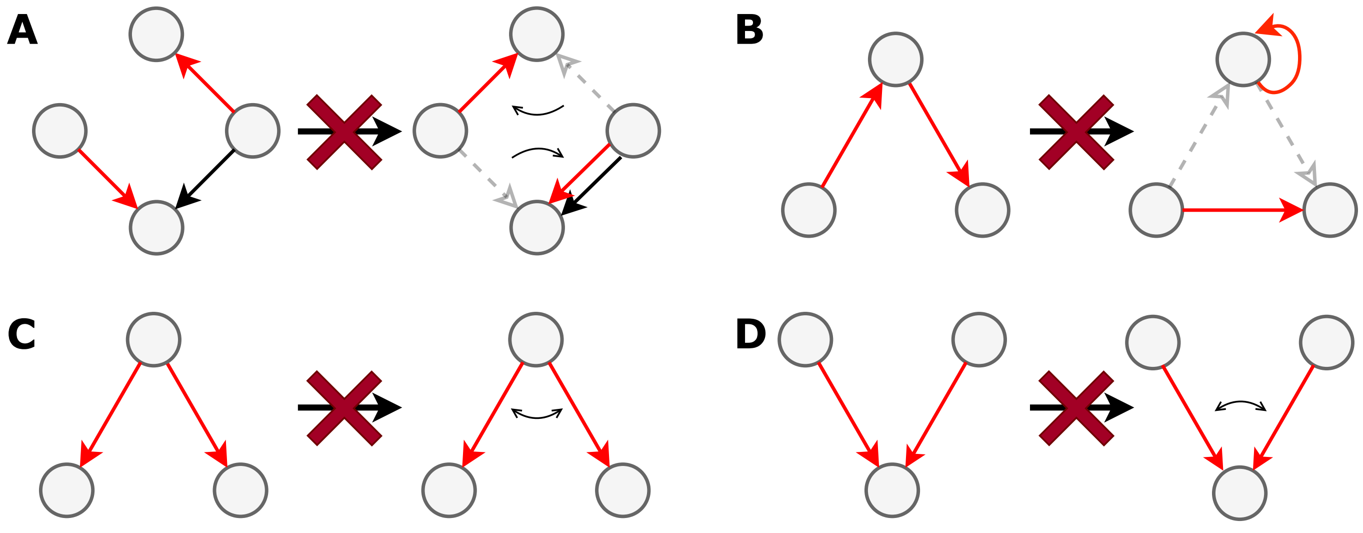

FIG. S2. Illustration of the pathological cases we want to avoid in the swap-rewiring algorithm. In panel **A**, we illustrate the potential introduction of a multiedge. The source in the first link should not be an in-neighbor of the target in the second one, or equivalently, the target in the first link should not be an out-neighbor of the source in the second one. Panel **B** shows the scenario that would introduce self-loops. Panels **C** and **D** represent two trivial swaps that do not change the topology in unweighted networks.

B. Sampling two links in the network

In each Monte Carlo step, two links in the network are rewired according to the scheme in Fig.2 in the main paper. For the case of unweighted networks, the two links are chosen in the following way. At the beginning of each rewiring event, the current network configuration is characterized by the set of nodes $\mathcal{N}$, which never changes, and the set of links $\mathcal{L}$. A first link l_1 is selected at random (with uniform probability), connecting $l_1^{source} \rightarrow l_1^{target}$. The second link l_2 should have the same combination of NACE 3-digit groups to avoid affecting the production functions of the nodes. This means that the source l_2^{source} must belong to the set of nodes with the same NACE3 code of l_1^{source}, i.e., $\mathcal{S} = \{n \in \mathcal{N} \mid \text{NACE3}(n) = \text{NACE3}(l_1^{source})\} \subset \mathcal{N}$. From this set, we further exclude:

- Node l_1^{target}, because otherwise we would introduce self-loops (see Figure S2 B)). The set is restricted to:

$$\mathcal{S}' = \mathcal{S} \setminus \{l_1^{target}\}$$

- For networks with binary links, in-neighbors of l_1^{target} are also excluded from the set of possible sources. Choosing node l_1^{source} again as the source in l_2 results in the trivial link swap between customers of the same firm in panel C). Choosing another in-neighbor and proceeding with the swap would introduce a link that already exists, resulting in increasing its weight by 1 (panel A)). For unweighted networks, the set of possible sources is then:

$$\mathcal{S}'' = \mathcal{S}' \setminus \{l_1^{source}\} \setminus \{n \rightarrow l_1^{target}\}$$

Similarly, the target in the second link should belong to $\mathcal{T} = \{n \in \mathcal{N} \mid \text{NACE3}(n) = \text{NACE3}(l_1^{target})\}$. Here, we exclude:

- node l_1^{source}, otherwise the introduction of self-loops (panel B)):

$$\mathcal{T}' = \mathcal{T} \setminus \{l_1^{source}\}$$

- For networks with binary links, out-neighbors of l_1^{source} are ruled out. Node l_1^{target} leads to ineffective link-swap between suppliers (panel D)), and choosing other out-neighbors of l_1^{source} increases link weights (panel A)). Finally, the target in l_2 should be picked from:

$$\mathcal{T}'' = \mathcal{T}' \setminus \{l_1^{target}\} \setminus \{l_1^{source} \rightarrow n\}$$

Every link pointing from a node in $\mathcal{S}''$ to a node in $\mathcal{T}''$ is eligible as a second link for the swap, so we intersect the links going out from nodes in $\mathcal{S}''$ with the links ending to nodes in $\mathcal{T}''$ and we choose one of them with uniform probability. In case the set is empty, the algorithm goes back to the first step and samples again the first link l_1.

In the case of weighted networks, the considerations that brought to the sets $\mathcal{S}'$ and $\mathcal{T}'$ are still valid, but there is no reason to forbid increasing the weight of a link as in panel A) and restricting to $\mathcal{S}''$ and $\mathcal{T}''$. Moreover, the swap of volumes between customers and suppliers becomes meaningful, as the node out-strengths are allowed to change if they stay between 80% and 120% of the empirical value. The second link is chosen by sampling the set of links from nodes in $\mathcal{S}'$ to nodes in $\mathcal{T}'$.

C. Pseudocode

We schematize the rewiring algorithm in the following pseudocode. In the unweighted rewiring, we want to avoid the introduction of multi-edges (increasing the weight of the links) and to save computational time, we also exclude the trivial swap of customers (suppliers) in the same firm. Lines 7 and 9 in the FIND2LINKS prevent these scenarios, depicted in Figure S2 , C) and D). After finding two links, procedure REWIRING handles the weighted-unweighted cases. The functions SWAPPARTIAL and SWAPFULL contain the operations on the two links discussed in detail in the first paragraph of this section.

```
 1: procedure FIND2LINKS(G)
 2:     while link2 = NULL do
 3:         link1 ← sample(edges(G))
 4:         source, target ← ends(link1)
 5:         SourceCandidates ← sameNACE3(G, source) \ target
 6:         SourceCandidates ← SourceCandidates \ inneighbors(target)      ▷ In case of unweighted networks
 7:         TargetCandidates ← sameNACE3(G, target) \ source
 8:         TargetCandidates ← TargetCandidates \ outneighbors(source)     ▷ In case of unweighted networks
 9:         OutgoingLinks ← edges(G).from(CandidateSources)
10:         IncomingLinks ← edges(G).to(CandidateTargets)
11:         bridges ← OutgoingLinks ∩ IncomingLinks
12:         link2 ← sample(bridges)
13:     end while
14:     return link1, link2
15: end procedure
16:
17: procedure REWIRING(G)
18:     while TRUE do
19:         link1, link2 ← FIND2LINKS(G)
20:         residue ← |weight(link1) − weight(link2)|
21:         if residue > threshold then
22:             SwappedG ← SWAPPARTIAL(G, link1, link2)
23:             break
24:         else if SUPPLIERS_OUTSTRENGTHS_CONSTRAINT(link1,link2) then
25:             SwappedG ← SWAPFULL(G, link1, link2)
26:             break
27:         end if
28:     end while
29:     return SwappedG
30: end procedure
```

FIG. S3. Procedure FIND2LINKS in the pseudocode summarizes the searching algorithm for choosing two links in the weighted or unweighted network G. Procedure REWIRING summarizes how to handle the cases for weighted or unweighted swap-rewiring.

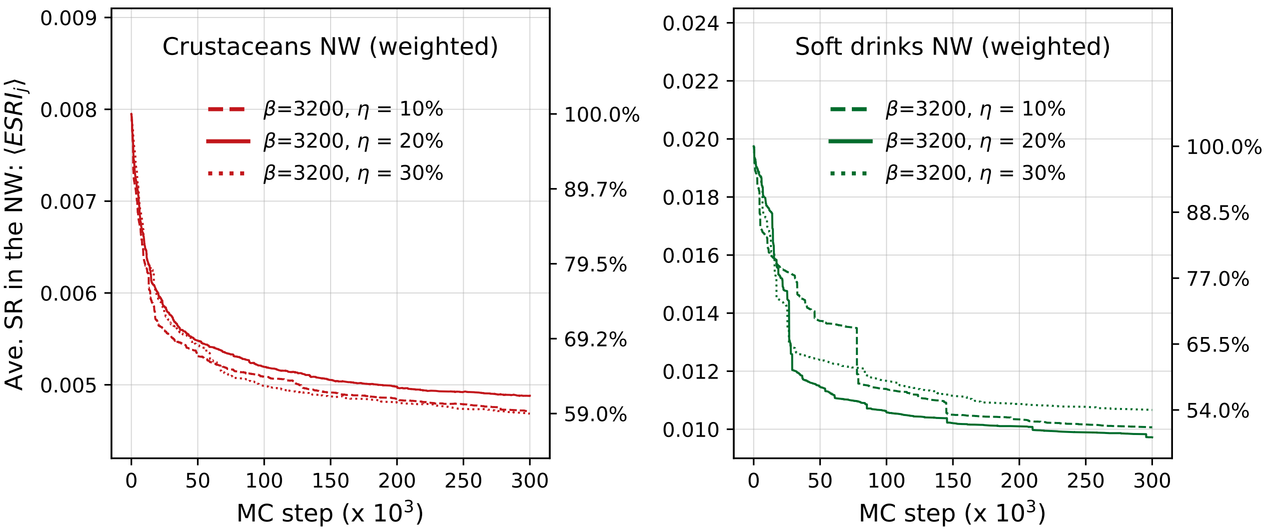

FIG. S4. Evolution of ⟨ESRI⟩ along the Monte Carlo simulations for the two weighted networks, Crustaceans and Soft drinks. For each network, the three rewiring simulations run with the same seed and value of β, but different values of the parameter η constraining the maximum tolerated change in out-strength of the suppliers when swapping two links with similar but not identical weight changes their out-strength. A lower (larger) value of η results in fewer (more) possible swaps and a smaller (larger) network configuration space.

D. Weighted network swap and tolerated change in firms' out-strength

As discussed in subsection A, rewiring a network by swapping two links must account for differences in link weights. Small changes in out-strength of source nodes can accumulate over thousands of rewiring events in a simulation, potentially leading to large deviations from empirically observed values and substantial distortions of firms' technical coefficients. To prevent this effect, we impose a constraint $\eta = 20\%$ on the tolerated change in the out-strength of the source nodes involved in a swap. Specifically, for any firm i, we require its out-strength to remain within a maximum deviation $\eta = 20\%$ from its empirical value $s_{emp}^{i,out}$. Thus, if the swap proposed at the Monte Carlo step t would result in an out-strength $s_{t+1}^{i,out}$ outside the interval $\left[(1-\eta)\ s_{emp}^{i,out}, (1+\eta)\ s_{emp}^{i,out}\right]$, the swap is rejected. The lowest possible value of η is 0, and corresponds to the case where rewiring does not affect the sources' strengths and the two swapped link weights have the same value.

We investigate the role of η by running rewiring simulations for systemic risk mitigation using the same fixed seed and value of inverse temperature β, but different values of parameter η. Using the same seed means that the random link couples candidated for the swaps are the same. Also the Metropolis-Hastings criterion for move acceptance $p = \min\{1, \exp(-\beta\Delta\langle \mathrm{ESRI}\rangle)\}$ accepts and rejects the same moves. The simulations can only diverge when the rewiring algorithm finds a couple of links that would change the out-strength of a node to a value that is accepted by one tolerance value, and rejected by the other ones.

In Figure S4 we show the systemic risk trajectories of rewiring simulations in the two weighted networks (Crustaceans and Soft drinks) using three different values of η: 10% (dashed line), 20% (solid line), and 30% (dotted line). A larger η increases the capacity of the source nodes to accommodate link swaps, expanding the space of possible network configurations, and potentially augmenting the achievable systemic risk mitigation. On the contrary, a lower η translates into a more stringent constraint for network rewiring, a smaller space of possible configurations, and should result in milder risk mitigation results. However, the risk mitigation achieved at $\eta = 10\%$ and 30% is in the same order of magnitude as the results at 20% (and $\beta = 3200$) in Figure S5, suggesting that the result is robust to changes in the value of η.

S4. RESULTS FOR ALL THE FIXED β AND SIMULATED ANNEALING MONTE CARLO SIMULATIONS

In the main text, we reported the results of three runs of the $\langle \mathrm{ESRI} \rangle$ minimization at fixed β and one simulated annealing run, for one weighted network (soft drinks) and one unweighted network (food production). Here, we attach the results for all the tested values of fixed β, for all the networks. In Figure S5, it is evident how the convergence value of $\langle \mathrm{ESRI} \rangle$ depends on the $\beta = 1/T$ temperature parameter. In each subfigure, only one trajectory per β is reported. The simulated annealing temperature curve was calibrated after the results with fixed beta, and the chosen curves are reported in Table S2. Non trivially, when comparing $\langle \mathrm{ESRI} \rangle$ values from the empirical data, those obtained from random explorations of the configuration space ($\beta = 0$), and the minimized states accessed through the Metropolis-Hastings algorithm, empirical networks are consistently closer to undriven random configurations than to the risk-mitigated states. This suggests that none of the analyzed networks can naturally reach the minimal $\langle \mathrm{ESRI} \rangle$ states without implementing policies that promote some rewiring behavior.

Network	Food production (unweighted)	Automotive (unweighted)	Crustaceans (unweighted)	Soft drinks (unweighted)	Crustaceans (weighted)	Soft drinks (weighted)
Simulated annealing β curve	$\beta(\mathrm{step}) = \frac{12800 \cdot \mathrm{step}}{50000}$	$\beta(\mathrm{step}) = \frac{12800 \cdot \mathrm{step}}{50000}$	$\beta(\mathrm{step}) = \frac{6400 \cdot \mathrm{step}}{200000}$	$\beta(\mathrm{step}) = \frac{2100 \cdot \mathrm{step}}{300000}$	$\beta(\mathrm{step}) = \frac{7200 \cdot \mathrm{step}}{300000}$	$\beta(\mathrm{step}) = \frac{3600 \cdot \mathrm{step}}{300000}$

TABLE S2. Temperature decrease curves used for the simulated annealing results.

S5. COMPARISON OF THE ESRI PROFILES IN THE BEST $\langle \mathrm{ESRI} \rangle$ MINIMIZATION RESULTS AND RELATIVE EMPIRICAL NETWORKS.

In Figure S6, S7 and S8, we report for each network the comparison between the ESRI attributes of the 100 riskiest nodes in the empirical data and their new values in the most effective minimization. In the main plot of each subfigure, the firms are ranked by their ESRI values in the empirical network along the x-axis, with only the riskiest 100 firms displayed. For each firm, ESRI in the empirical network (lighter color bars) is compared to ESRI in the risk-mitigated network. In the inset, firms are re-ordered by their ESRI values in both the empirical and mitigated networks, allowing for a direct comparison of the distribution profiles. This means that a firm's rank in the empirical network (lighter line) may differ from its rank in the mitigated network (black line).

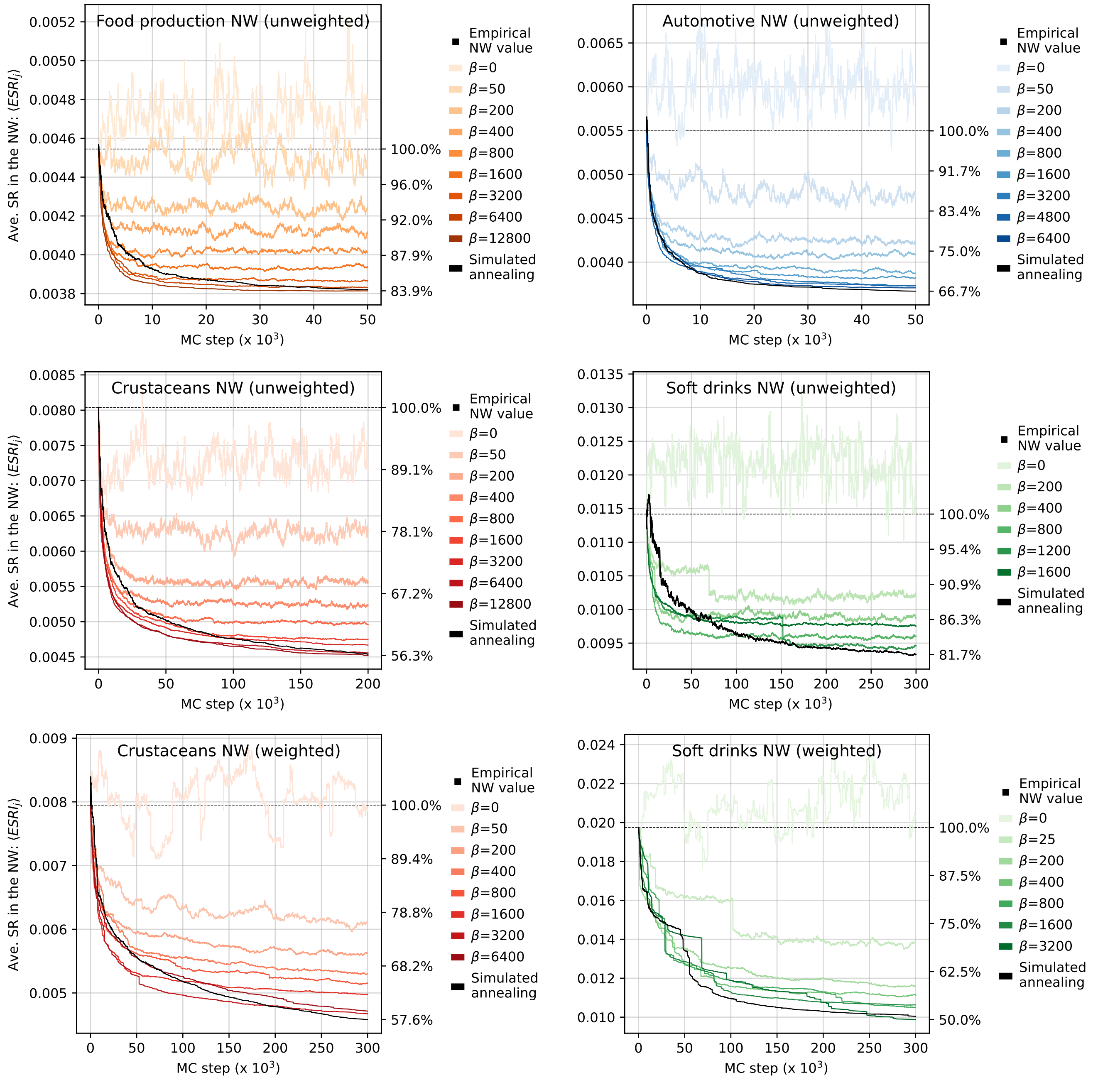

FIG. S5. Evolution of ⟨ESRI⟩ along the Monte Carlo simulations for all the networks. The first two rows display results for unweighted networks, while the last row shows those for weighted networks. Each subfigure includes a trajectory from simulated annealing and one trajectory for each fixed β value tested. Lowering the temperature ($T = 1/\beta$) generally decreases the convergence value, although excessively low T can cause the exploration to get stuck. The fixed-β results were used to calibrate the parameters for the simulated annealing procedures of Table S2.

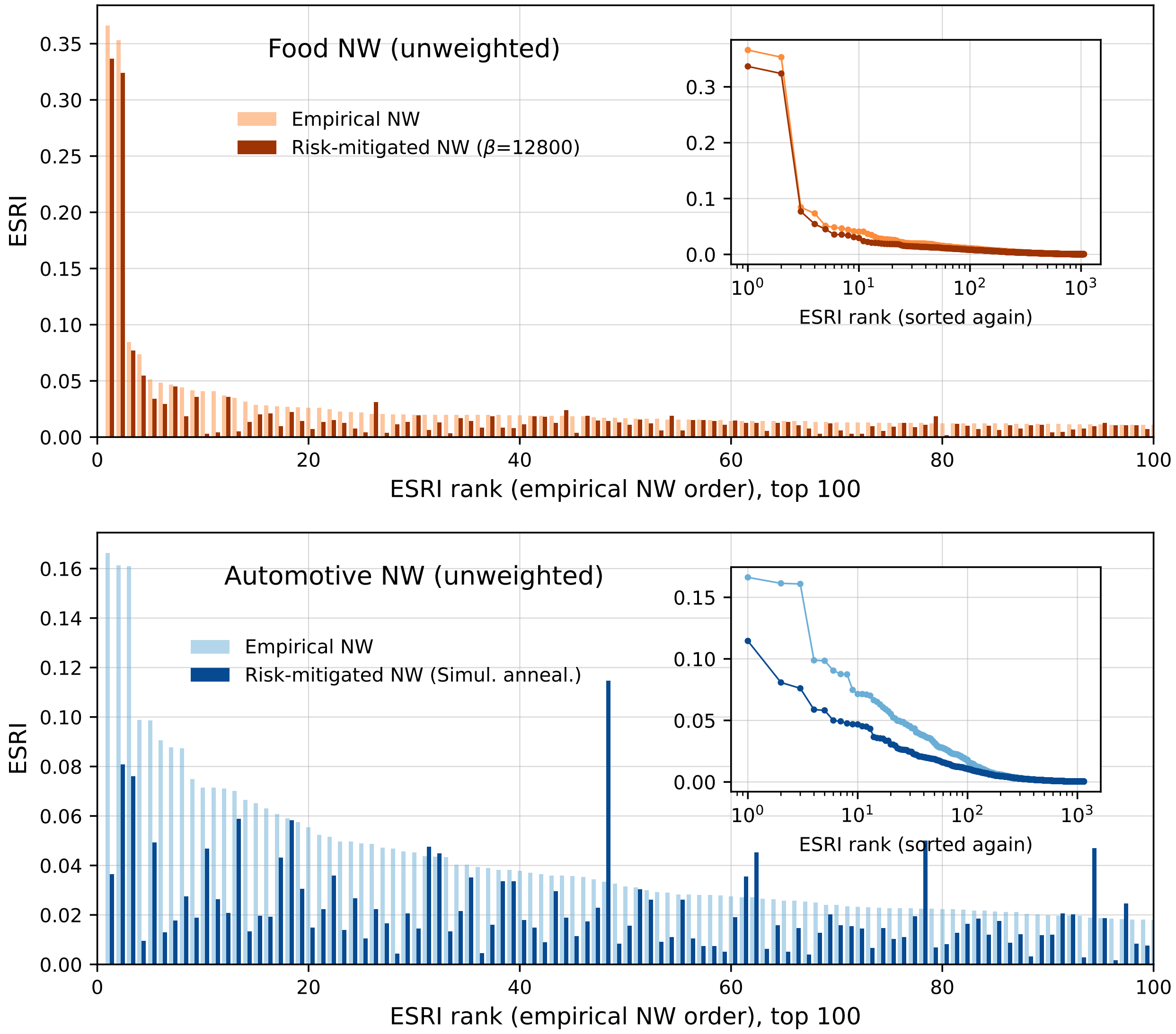

FIG. S6. Systemic risk profiles of the food production network (top panel) and automotive network (bottom panel). The networks are considered in their unweighted version. The light bars report firms' ESRI values in the empirical network, the darker bars the new ESRI values in the rewired networks. We consider the best risk-mitigation results with the largest reduction of $\langle \text{ESRI} \rangle$, fixed $\beta = 12800$ and simulated annealing. Firms are ranked by their ESRI in the empirical network along the x-axis, displaying only the top 100 riskiest firms. The inset presents the same profiles, with the rewired profile now rank-ordered for direct comparison.

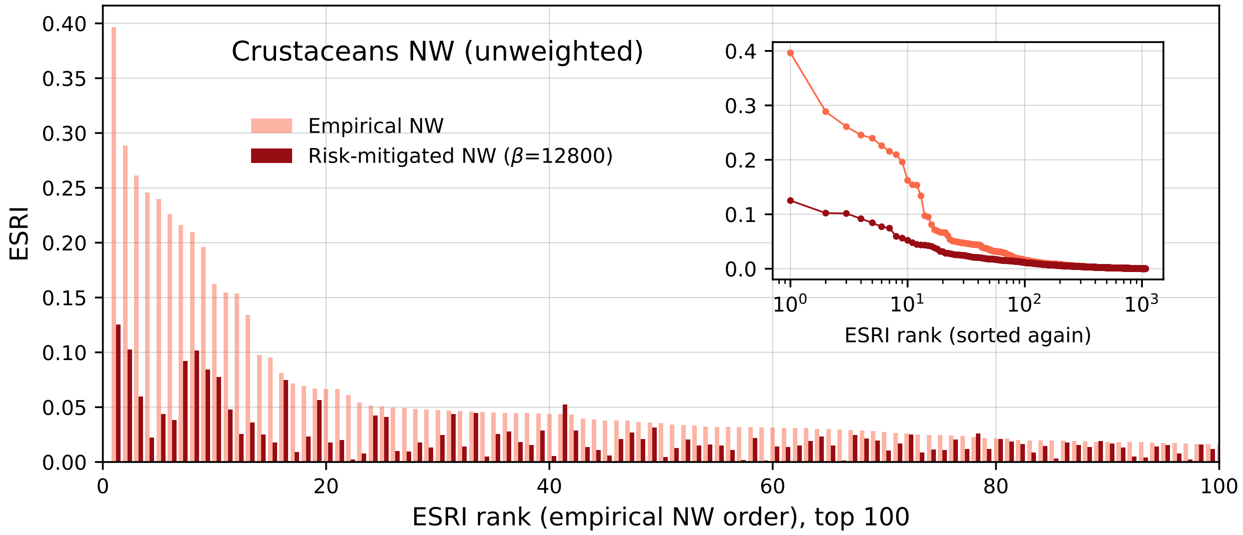

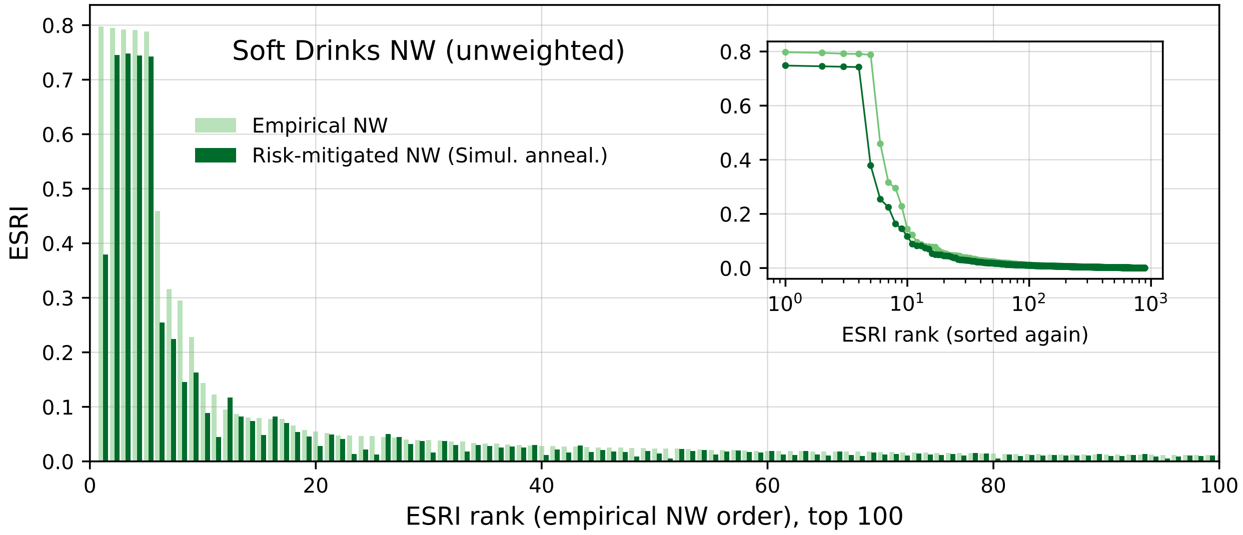

FIG. S7. Systemic risk profiles of the crustacean network (top panel) and soft drink network (bottom panel). The networks are considered in their unweighted version. The light bars report firms' ESRI values in the empirical network, the darker bars the new ESRI values in the rewired networks. We consider the best risk-mitigation results with the largest reduction of $\langle \text{ESRI} \rangle$, fixed $\beta = 12800$ and simulated annealing. Firms are ranked by their ESRI in the empirical network along the x-axis, displaying only the top 100 riskiest firms. The inset presents the same profiles, with the rewired profile now rank-ordered for direct comparison.

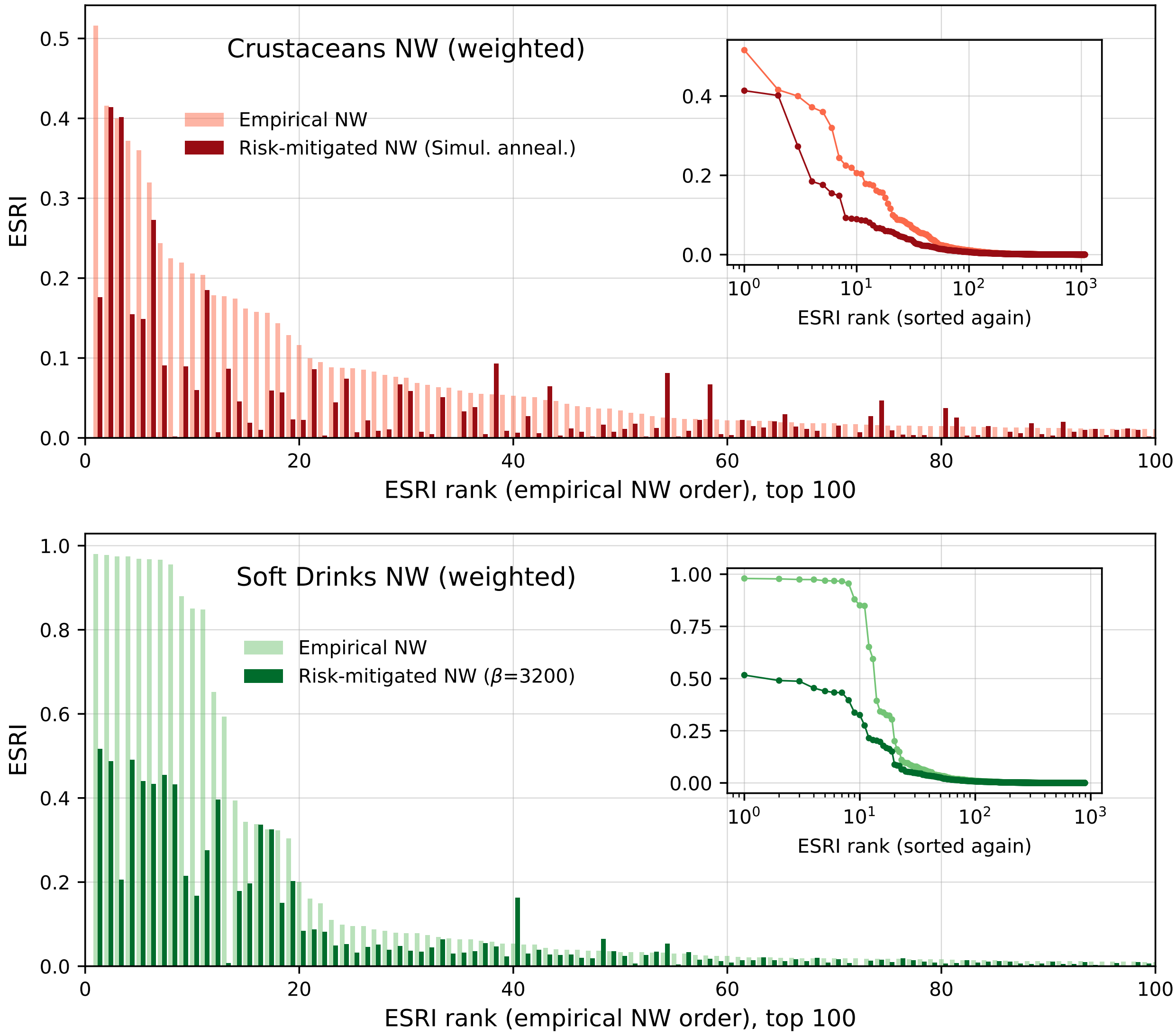

FIG. S8. Systemic risk profiles of the crustacean network (top panel) and soft drink network (bottom panel). The networks are considered in their weighted version. The light bars report firms' ESRI values in the empirical network, the darker bars the new ESRI values in the rewired networks. We consider the best risk-mitigation results with the largest reduction of ⟨ESRI⟩, simulated annealing and fixed $\beta = 3200$. Firms are ranked by their ESRI in the empirical network along the x-axis, displaying only the top 100 riskiest firms. The inset presents the same profiles, with the rewired profile now rank-ordered for direct comparison.

S6. RANDOM EXPLORATION OF THE CONFIGURATION SPACE ($\beta = 0$)

We compared the empirical networks with the ones resulting from the $\langle$ESRI$\rangle$ minimization considering several network measures, and reported the results in Table 1 in the main text. Some of the measures are different before and after the minimization process. Their different final values may be distinctive characteristics of the more robust network states, or simply an emergence of the configuration model. To understand if any of these changes carries information about the increased robustness, we rewired the networks with the same algorithm without considering the driving Metropolis-Hastings criterion, and so accepting every link rewiring.

For each network measure that has changed with the $\langle$ESRI$\rangle$ minimization, we compared the measure's trajectory in the non-driven rewiring simulation, considering the network state every 5000 Monte Carlo steps, and the measure's punctual value in the network with the best $\langle$ESRI$\rangle$ minimized result. In the weighted Ecuadorian networks, we considered the number of links L, the reciprocity of the links, the average total degree $\langle k^{tot}\rangle$, the mean average neighbors' total degree $\langle\langle k^{tot}\rangle_{NN}\rangle$, the average local clustering coefficient, and the size of the largest strongly connected component. The results are reported in Figure S9, with one network measure per subfigure and trajectories colored after the considered network. The black points represent the $\langle$ESRI$\rangle$ minimized networks. Looking in particular at the first subfigure in Figure S9, the increased number of links reaches similar values in the driven and non-driven rewiring. This means that L does not explain the decrease in systemic risk in the risk-mitigated network, which informs us that the robustness does not come from just diversifying the number of suppliers or customers. Because the unweighted networks do not change the number of links, we considered only reciprocity, mean average neighbors' total degree $\langle\langle k^{tot}\rangle_{NN}\rangle$, average local clustering coefficient, and size of the largest strongly connected component. Results are plotted in Figure S11 (Ecuador) and S10 (Hungary).

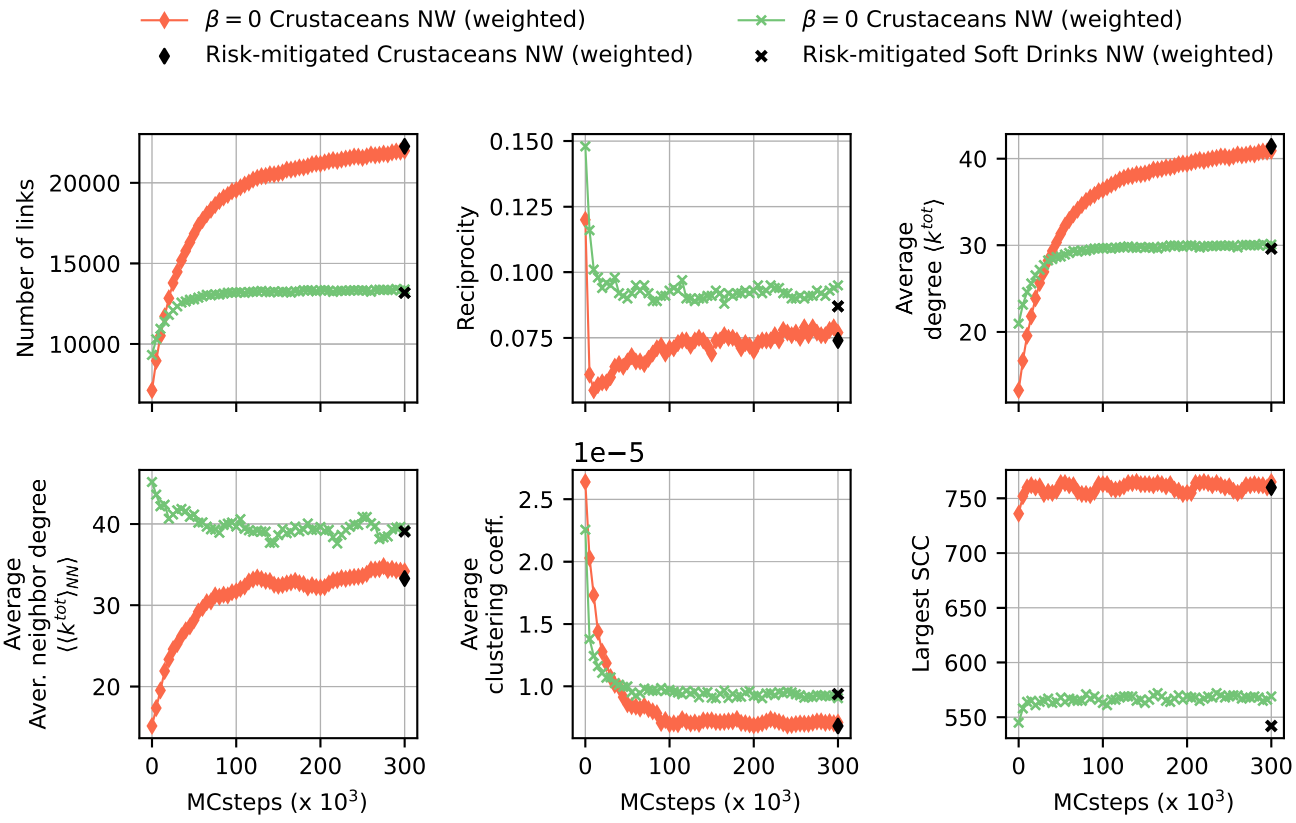

FIG. S9. Evolution of several network measures for the configuration model. Link rewiring is always accepted, without evaluating $\Delta\langle$ESRI$\rangle$ in the Metropolis-Hastings criterion. In each subfigure, for each weighted Ecuadorian network, the trajectory of the measure is compared with the final value of the simulation minimizing $\langle$ESRI$\rangle$.

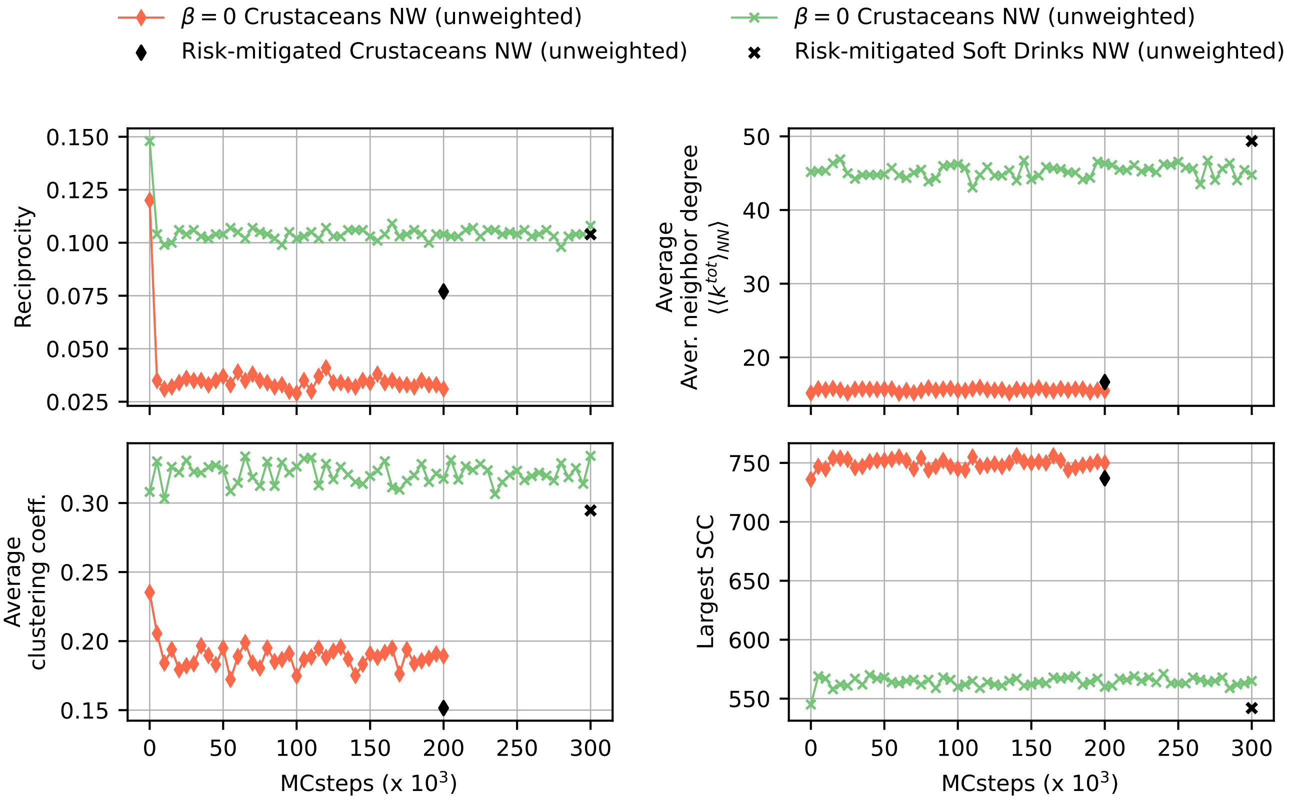

FIG. S10. Evolution of several network measures for the configuration model. Link rewiring is always accepted, without evaluating $\Delta\langle \mathrm{ESRI}\rangle$ in the Metropolis-Hastings criterion. In each subfigure, for each unweighted Ecuadorian network, the trajectory of the measure is compared with the final value of the simulation minimizing $\langle \mathrm{ESRI}\rangle$.

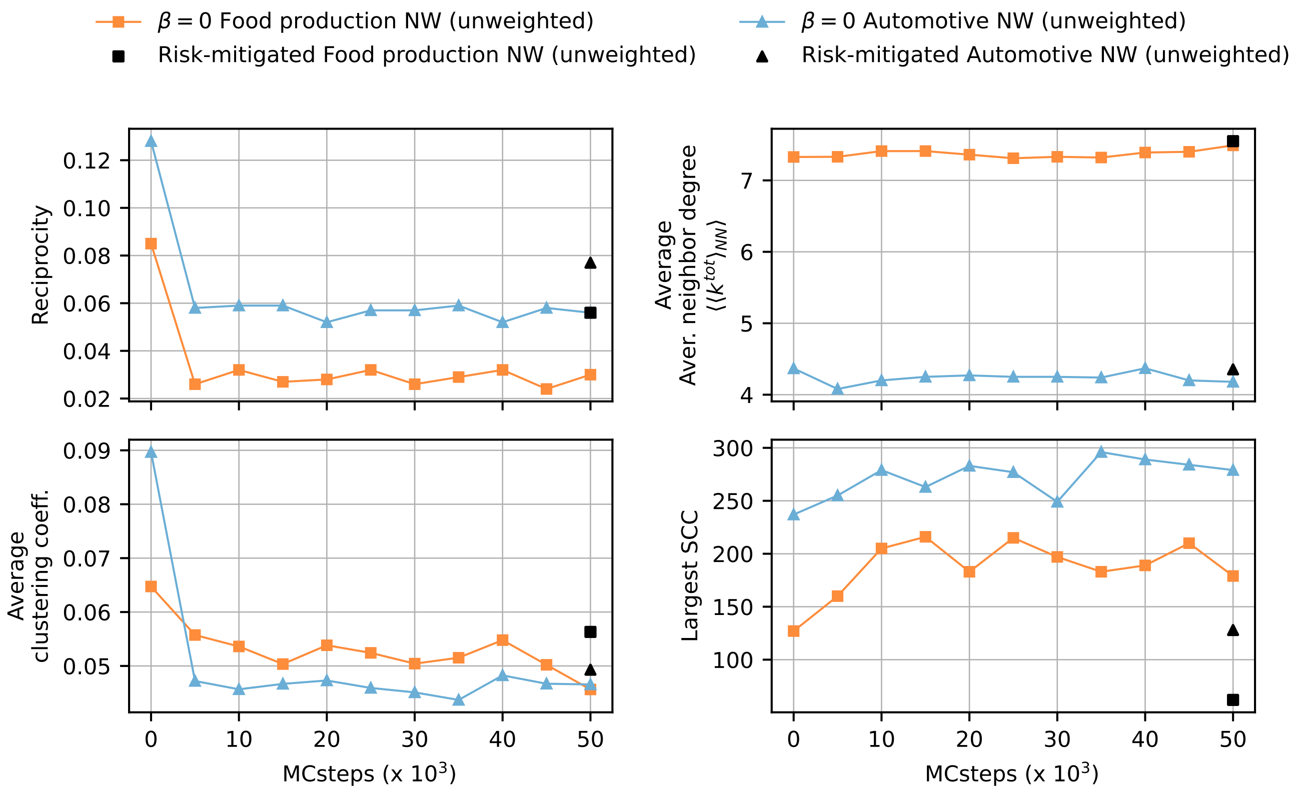

FIG. S11. Evolution of several network measures for the configuration model. Link rewiring is always accepted, without evaluating $\Delta\langle \mathrm{ESRI}\rangle$ in the Metropolis-Hastings criterion. In each subfigure, for each unweighted Hungarian network, the trajectory of the measure is compared with the final value of the simulation minimizing $\langle \mathrm{ESRI}\rangle$.

S7. FIRMS' CONNECTIVITY AND SYSTEMIC RISK CONTRIBUTION

Since a firm's ESRI is determined by the cascading production failures triggered when its supply orders and output deliveries are halted, it heavily depends on the relevance of the firm's connections to the production of its supply chain neighbors. Consequently, firms with high in, out, or total strength - whether large hubs connected to many firms or those with only a few but highly weighted links - tend to trigger larger cascades and have high ESRI. A key finding of [5] is that even small yet essential suppliers to large companies can carry significant systemic risk. In unweighted networks, where all links are treated equally, node strength corresponds to node degree. We analyze the relationship between firms' connectivity and ESRI in our subnetworks and report in Fig. S12 and S13 the scatterplots of node strength or degree against ESRI (in log-log scale). For each row, the subfigure on the left and on the right show the results for the empirical production network and for the best result of the risk-mitigation rewiring. For unweighted networks, the rewiring algorithm preserves firms' degree, but alters their neighborhood. While degree values remain unchanged, ESRI values tend to decrease after rewiring. The correlation between degree and ESRI is evident, and rewiring makes the scatterplot points more concentrated, with a more linear dependence (in log-log scale). For weighted networks, ESRI appears to exhibit a stronger linear dependence on node strength (again, in log-log scale). Unlike in [5], where many small firms (low strength) were found to have high systemic importance in the Hungarian SCN, our analysis does not show this pattern clearly. We attribute this to the economic activity (NACE *groups*) filtering process, which may have excluded these small-size suppliers of essential goods for large companies. However, firms with strengths several orders of magnitude smaller than the largest firms but comparable ESRI values are still present.

Lastly, while a firm's node degree remains unchanged between weighted and unweighted network representations, its ESRI can vary due to the different technological coefficients for firm production. The availability of weight information increases the level of detail in modeling the relevance of a firm for its suppliers and customers, and thus the potential cascade of failures through its network neighbors.

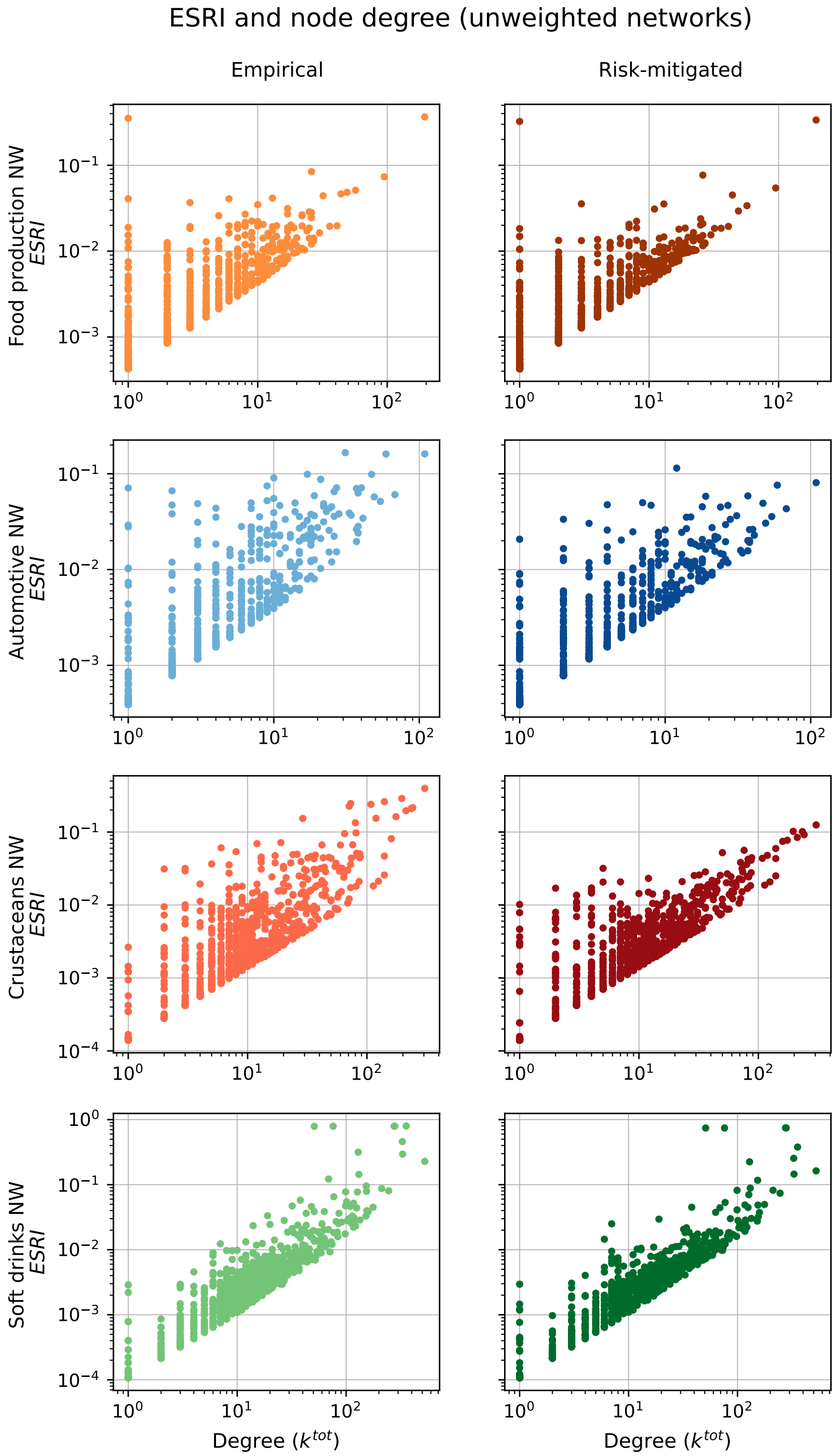

FIG. S12. Relationship between firms' Economic Systemic Risk Index (ESRI) and node total degree (k^{tot}) across the four unweighted networks. Each row corresponds to a distinct network: Food production, Automotive, Crustaceans, and Soft drinks. The left column shows results for empirical networks, while the right column presents risk-mitigated networks. For unweighted networks, the rewiring algorithm reduces the overall risk magnitudes without affecting node degrees. The plots highlight the positive correlation between node degree and ESRI. A high degree often indicates high systemic importance, but the reverse is not always true. Axes are displayed on logarithmic scales.

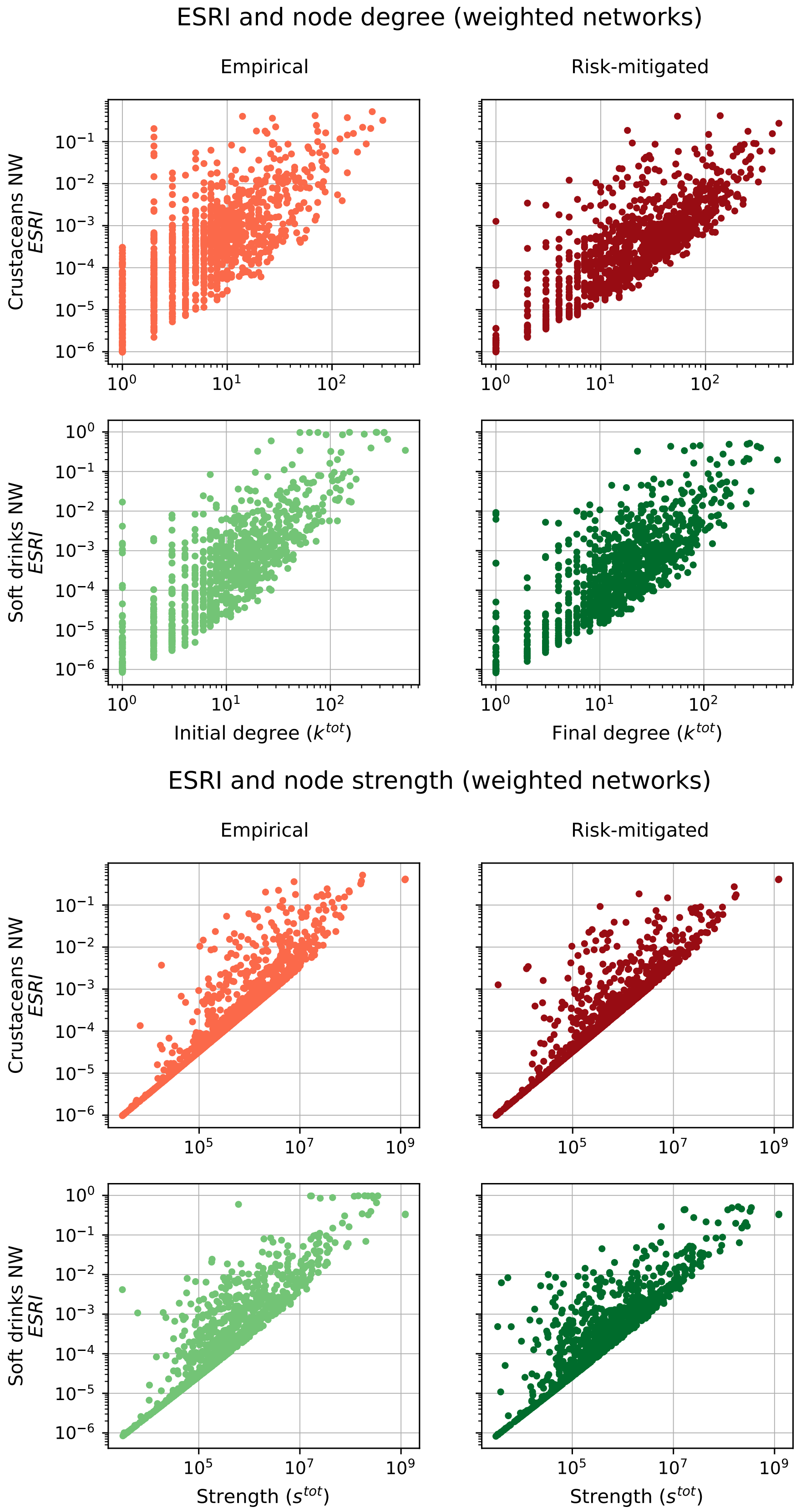

FIG. S13. Relationship between firms' Economic Systemic Risk Index (ESRI), node total degree (k^{tot}) and total strength (s^{tot}) across the two weighted networks. Each row corresponds to a distinct network: Crustaceans, and Soft drinks. The left column shows results for empirical networks, while the right column presents risk-mitigated networks. For weighted networks, the rewiring algorithm reduces the overall risk magnitudes, preserving the strength per sector and occasionally changing node degrees.

S8. WEIGHTED NETWORKS REWIRING AND RELATIONSHIP BETWEEN CHANGE IN NODE DEGREE AND ESRI

The rewiring algorithm can alter the number of links L in the network, either when the two links selected for swapping have significantly different weights (see Fig. 2 in the main text) or when the cross-links already exist (see Fig. S2 A)). Since, in practice, the rewiring process in weighted networks substantially increases the number of links (see Tab.1 in the main text), we investigate whether the observed systemic risk mitigation results from diversification—i.e., firms gaining more suppliers and customers. The simulation results at $\beta = 0$ already demonstrate that simply increasing L is not a sufficient condition for risk mitigation. At this parameter value, L is comparable to that in the best risk-mitigation configurations, yet the average systemic risk $\langle \text{ESRI} \rangle$ remains at empirical levels. In this section, we examine to what extent increasing L is a necessary condition for risk mitigation.

We focus on the best risk-mitigation results in the weighted crustaceans and soft drinks production networks, comparing the changes in node degree k^{tot} and ESRI in the networks before and after the rewiring simulation. The top panel of Figure S14 presents scatterplots showing how each node's k^{tot} and ESRI value changed, in terms of absolute Δ. While increasing k^{tot} generally reduces ESRI (evidenced by more points in the bottom right than the bottom left), many firms decrease their ESRI without a significant change in k^{tot}. Notably, in the soft drinks network in particular, some firms even concentrate their transactions on fewer partners ($\Delta\text{ESRI} < 0$, $\Delta k^{tot} < 0$). This indicates that diversification is not strictly necessary for individual firms to reduce systemic risk.

On a broader scale, the network undergoes a structural reorganization where increasing the number of connections for some nodes can reduce—or even increase—ESRI for others. However, relatively few firms experience an increase in ESRI (region at $\Delta\text{ESRI} > 0$), which aligns with our observations from Fig. 4 in the main text: while a few outliers increase their ESRI, the majority of the firms experience a reduction. One possible scenario could have been that the riskiest firms redistribute their systemic importance across smaller firms, leading to a lower average ESRI at the cost of shifting the tail of the ESRI distribution upward. However, this does not appear to be the case.

Because node strength and degree strongly correlate with systemic importance (see Figure S13) and follow a fat-tailed distribution, not all firms play a major role in systemic risk mitigation. This results in a dense cluster of points around $\Delta\text{ESRI} = 0$. We focus on firms that significantly contributed to risk mitigation by reducing their ESRI by at least 0.01, as the self-organization of high-ESRI firms towards a more resilient network may follow different dynamics. The bottom panel of Fig. S14 shows the relative changes in degree and ESRI. A vertical line distinguishes regions where firms had relatively lower (< 1) or higher (> 1) degrees after rewiring. Even for these larger contributors, the pattern seems the same: increasing the degree generally reduces ESRI, but some firms achieved a 50% ESRI reduction despite a lower degree after rewiring (soft drinks network).

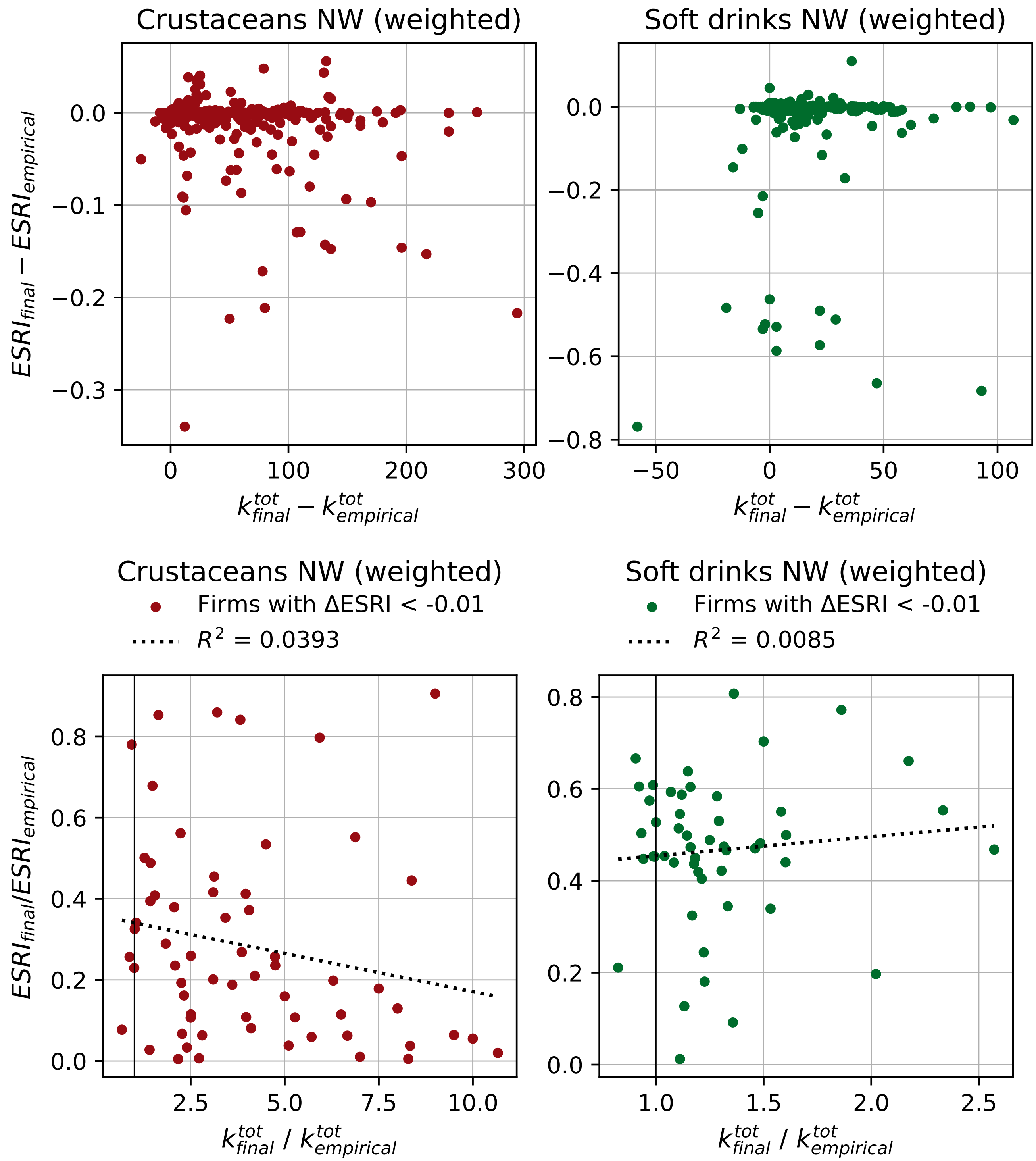

FIG. S14. Scatterplot Δ k against Δ ESRI (all nodes), and scatterplot with the relative k change against relative ESRI change focusing the firms that have a significant Δ ESRI.

[1] International Standard Industrial Classification of All Economic Activities Revision 4, Series M: Miscellaneous Statistical Papers, No. 4 Rev. 4, New York: United Nations. ST/ESA/STAT/SER.M/4/REV.4 (2008).

[2] Regulation (EC) No 1893/2006 of the European Parliament and of the Council of 20 December 2006 establishing the statistical classification of economic activities NACE Revision 2 and amending Council Regulation (EEC) No 3037/90 as well as certain EC Regulations on specific statistical domains Text with EEA relevance (2006).

[3] The Growth Lab at Harvard University, The Atlas of Economic Complexity (2013), `http://www.atlas.cid.harvard.edu.`

[4] A. Pichler, M. Pangallo, R. M. del Rio-Chanona, F. Lafond, and J. D. Farmer, Forecasting the propagation of pandemic shocks with a dynamic input-output model, Journal of Economic Dynamics and Control **144**, 104527 (2022).

[5] C. Diem, A. Borsos, T. Reisch, J. Kertész, and S. Thurner, Quantifying firm-level economic systemic risk from nation-wide supply networks, Scientific Reports **12**, 7719 (2022).
